# Transcriptome Analysis Reveals Multiple Hormones, Wounding and Sugar Signaling Pathways Mediate Adventitious Root Formation in Apple Rootstock

**DOI:** 10.3390/ijms19082201

**Published:** 2018-07-27

**Authors:** Ke Li, Yongqi Liang, Libo Xing, Jiangping Mao, Zhen Liu, Feng Dong, Yuan Meng, Mingyu Han, Caiping Zhao, Lu Bao, Dong Zhang

**Affiliations:** 1 College of Horticulture, Northwest A&F University, Yangling 712100, China; keli505@nwafu.edu.cn (K.L.); libo_xing@nwafu.edu.cn (L.X.); mjp588@nwafu.edu.cn (J.M.); zlhieun@nwafu.edu.cn (Z.L.); dong-feng@nwafu.edu.cn (F.D.); myhh0430@nwafu.edu.cn (Y.M.); hanmy@nwsuaf.edu.cn (M.H.); zhcc@nwsuaf.edu.cn (C.Z.); baolu@nwsuaf.edu.cn (L.B.); 2 Beijing Ori-Gene Science and Technology Corp., Ltd., Beijing 100000, China; liangyq513@126.com

**Keywords:** apple rootstock, transcriptome, adventitious root (AR), hormones, indole-3-butyric acid (IBA), sugar metabolism, wounding, cell cycle

## Abstract

Adventitious roots (AR) play an important role in the vegetative propagation of apple rootstocks. The potential role of hormone, wounding, and sugar signalling pathways in mediating AR formation has not been adequately explored and the whole co-expression network in AR formation has not been well established in apple. In order to identify the molecular mechanisms underlying AR formation in ‘T337’ apple rootstocks, transcriptomic changes that occur during four stages of AR formation (0, 3, 9 and 16 days) were analyzed using high-throughput sequencing. A total of 4294 differentially expressed genes were identified. Approximately 446 genes related to hormones, wounding, sugar signaling, root development, and cell cycle induction pathways were subsequently selected based on their potential to be involved in AR formation. RT-qPCR validation of 47 genes with known functions exhibited a strong positive correlation with the RNA-seq data. Interestingly, most of the candidate genes involved in AR formation that were identified by transcriptomic sequencing showed auxin-responsive expression patterns in an exogenous Indole-3-butyric acid (IBA)-treatment assay: Indicating that endogenous and exogenous auxin plays key roles in regulating AR formation via similar signalling pathways to some extent. In general, AR formation in apple rootstocks is a complex biological process which is mainly influenced by the auxin signaling pathway. In addition, multiple hormones-, wounding- and sugar-signaling pathways interact with the auxin signaling pathway and mediate AR formation in apple rootstocks.

## 1. Introduction

Apple (*Malus domestica* Borkh.) is one of the most commercially important fruits in the world. In terms of nutritional value and economic importance, apple, grape, orange, and banana are the most predominant fruit crops globally. Among these four fruit crops, apple is the most widely consumed and China is the world’s largest apple producer. Apple rootstocks play an important role in regulating the environmental adaptability and growth management of apple trees. The ‘T337’ dwarfing apple rootstock is widely used and confers early fruiting and high yields. Although the development of dwarfing, disease-resistant apple rootstocks is a major pursuit of the global apple industry, apple rootstock breeding programs in China have not been well established [1]. Adventitious root (AR) formation, however, represents a limiting factor in the vegetative propagation of apple rootstocks and other tree species. Unfortunately, the molecular mechanisms underlying adventitious rooting are still not completely understood. Thus, the study of the molecular regulatory mechanisms involved in AR formation in ‘T337’ apple rootstock is particularly important for understanding and solving the problems associated with AR development.

AR formation is a complex regeneration process that is influenced by many internal and external factors. ARs are distinct from lateral roots in that they form tissue that is not a root, such as stems and leaves, naturally or in response to altered environmental conditions [2,3]. Some studies have indicated that ARs are formed from cells in the pericycle of non-roots, which need to be established in order to initiate a root developmental program [4]. This process illustrates the enormous plasticity of plants and provides the basis for the practice of clonal propagation; a technology that is utilized for the production of most economically important horticultural crops. AR formation from stem cuttings is usually divided into several stages based on physiological and metabolic markers, and can be categorized as a four-stage process: Stage 1—the activation of cells in response to intrinsic signals; Stage 2—the reactivation of the cell cycle, which leads to the formation of an AR primordium; Stage 3—the activation of the root primordium (cell division and cell enlargement), and; Stage 4—outgrowth of the AR [5].

Plant hormones play an important role in the regulation of AR formation, as they modulate cell physiology in response to changes in the environment, provide a signaling network within the plant and determine cell fate and cell specialization. In many plant species, high auxin (AUX) levels in the basal region of stem cuttings are required for competent cells in the cambium to resume proliferation and to initiate a root-specific developmental program [6,7,8,9]. In addition, cytokinin (CTK), gibberellic acid (GA), abscisic acid (ABA), brassinolide (BR), and ethylene (Eth) have also been reported to mediate root formation [10,11,12,13,14]. The control and function of plant hormone homeostasis and related signaling networks in AR formation in apple rootstocks, however, is still not entirely understood. Interestingly, a stable relationship between AUX/ABA and AUX/CTK with AR formation has been reported, in which a high ratio of IAA/ABA and AUX/CTK promotes AR formation [15]. In contrast, the role of other hormone ratios in AR formation in apple rootstock, such as AUX/GA, GA/CTK, GA/ABA, ABA/AUX, and ABA/CTK is poorly understood.

Prior studies have suggested that auxin is the main hormone associated with AR formation [8,16,17,18]. As a result, exogenous application of IBA (Indole-3-butyric acid) is widely used to induce AR in many plant species. Several studies have identified an association between the expression of auxin-related genes and AR formation, including influx (Auxin Resistant 1/Like AUX-*AUX/LAX*) and efflux (PIN-Formed-*PIN*) carriers that play a role in the basipetal transport of auxin; allowing for the local accumulation of auxin at the stem base [8]. The auxin response factors, *ARF6* (AUXIN RESPONSE FACTOR 6) and *ARF8* (AUXIN RESPONSE FACTOR 8), have been identified as positive regulators of AR formation in *Arabidopsis* [19]. A variety of molecular and genetic approaches have been used to identify genes involved in regulating AR development in *Arabidopsis* and other plants. Several gene families in the auxin-related pathway are among the genes that have been shown to mediate adventitious rooting in many plants. The identified auxin-related genes include members of the auxin inducible *ARF* family [19,20], members of the auxin-inducible *GH3* (GRETCHEN HAGEN 3) family [20], members of indole-3-acetic acid (IAA) biosynthesis *YUCCA* gene family [21], the auxin efflux carrier genes, *PIN* (PIN-FORMED) and *ABCB/PGP* (ATP BINDING CASSETTE-TYPE B/P-GLYCOPROTEINs) [22,23,24], and the auxin influx carrier gene *AUX1/LAX* (AUXIN/IAA) [22,23]. Although many studies have documented the importance of auxin and auxin-related gene expression in the regulation of AR, the molecular signaling network involved in this process needs to be further detailed, especially in non-model species [25]. For example, in apple rootstocks, the role of these genes and gene families in AR formation is poorly understood.

While extensive research on a variety of physiological aspects of apple rootstocks has also been conducted, characterization of the underlying biochemical and cellular mechanisms responsible for the regulation of root regeneration is lacking. Therefore, in order to further understand the molecular mechanism of AR formation in apple rootstocks, a transcriptomic approach was used in the present study to identify genes associated with AR formation in ‘T337’ apple rootstocks. A high throughput sequencing analysis was used to provide the first global monitoring of changes occurring at the gene expression and whole co-expression network levels during AR formation in ‘T337’ apple rootstocks. The results obtained in the present study contribute to our basic understanding of the molecular events underlying AR formation in apple rootstocks. A perspective on future research on AR formation in tree species is also provided.

## 2. Results

### 2.1. Morphological Observations of the Process of AR Formation in Stem Cuttings of ‘T337’ Apple Rootstocks

**Figure 1:**
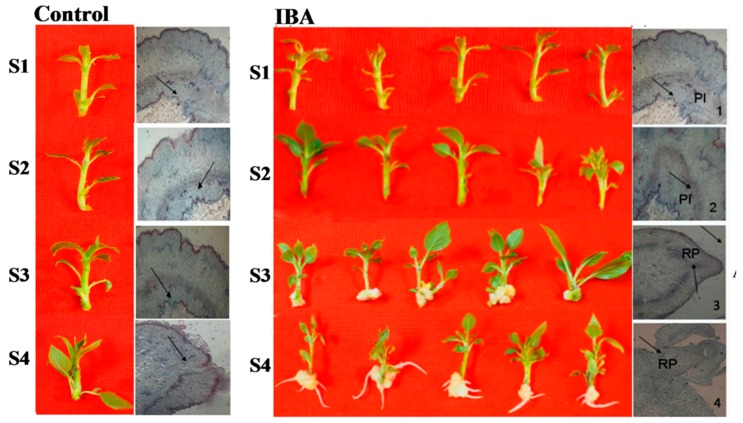
Time course and anatomical structure of AR formation in stem cuttings of ‘T337’ apple rootstocks. Time course of AR formation: S1 (stage 1) indicates the initial placing of the cuttings into rooting medium (0 days); S2 (stage 2) indicates that the cuttings were in the root medium for 3 days; S3 (stage 3) indicates that the cuttings were cultured in the rooting medium for 9 days; S4 (stage 4) indicates that the cuttings were cultured in the rooting medium for 16 days. Anatomical changes in stem cuttings of apple rootstock during AR formation. Stem cuttings of apple rootstock did not exhibit changes in the control. Under IBA-treatment (1) Young stem cross-section at 0 days (×60), PI (primordium initiation) indicates primordium initiation site; (2) evidence of vigorous cambium cell division at 3 days (×60), PI indicates primordium initiation site; (3) AR primordia are visible at 9 days (×40), RP (root primordium) indicates root primordium site; (4) elongation and outgrowth of ARs elongated at 16 days (×60), RP (root primordium) indicates root primordium site. Left and right sides of the figure represent control and IBA treatments, respectively. The positions pointed by arrows represents the region of AR primordium formation.

The ‘T337’ apple rootstock cultivar was chosen for this study due to its rooting performance relative to other rootstock genotypes, and its differential response to a mild IBA treatment during AR rooting (Figure 1). In the control treatment, ‘T337’ apple rootstocks did not exhibit any of the four stages of AR formation (Figure 1).

Under IBA-treatment, however, the ‘T337’ apple rootstocks formed callus and divided into roots in the fourth stage (Figure 1). The current study used an anatomical point-of-view analysis for the different stages of AR formation. Anatomical observations of AR formation were made by sectioning paraffin-embedded samples and viewing the stem sections through a light microscope. These observations confirmed the four stages of AR formation (Figure 1). Under IBA-treatment, the phenotype at S1 (0 days) and S2 (3 days) did not exhibit any significant changes (Figure 1); after 9 days of culturing in the rooting medium, callus tissue was clearly evident at the base of the stem (Figure 1), and anatomically, dome-shaped AR primordium were visible (Figure1); thus confirming the S3. At 16 days, AR emergence from the callus tissue was evident both morphologically and anatomically (Figure 1). However, under the control conditions, the stem tissue structure at the four stages did not exhibit any significant changes (Figure 1). Based upon the observations, AR formation can be divided into three phases: induction (S1 to S2); initiation (S2 to S3); and extension (S3 to S4). Collectively, these observations confirm the appropriateness of the sampling times that were used for the transcriptome profiling.

### 2.2. Quantitative Analyses of Hormone Levels in ‘T337’ Stem Cuttings during AR Formation

**Figure 2:**
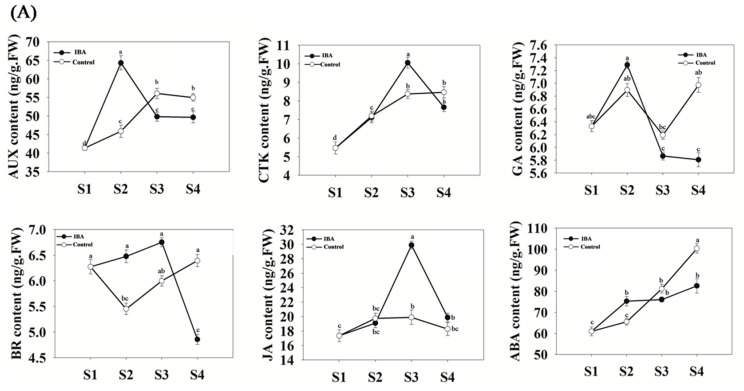

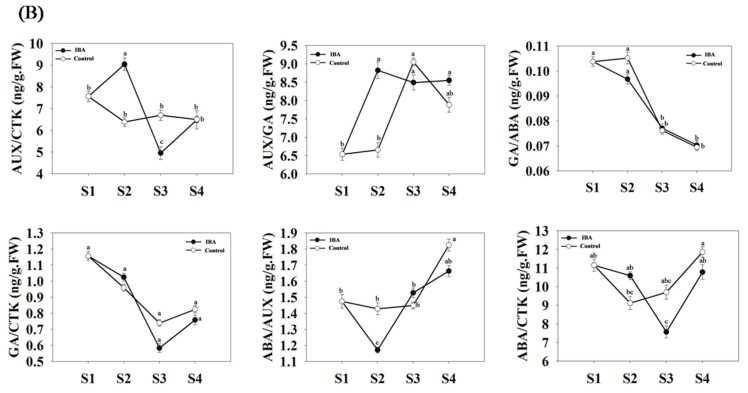
Hormone content and the hormone ratios in the basal sections (rooting zone) of stem cuttings of ‘T337’ apple root stocks during AR formation. (**A**) AUX: Auxin; CTK: cytokinin (zeatin riboside); GA: gibberellic acid 3; BR: brassinolide; JA: jasmonic acid; ABA: abscisic acid. (**B**) Ratios of hormone contents: AUX/CTK, AUX/GA, GA/CTK, GA/ABA, ABA/AUX, and ABA/CTK. Statistically significant differences in IBA-treated cuttings and control on each stages are indicated with different letters (a, b, c, ab, abc).

Hormone levels and their ratios to each other were analyzed in ‘T337’ cuttings at four time points during AR formation (Figure 2A,B). Under IBA-treatment, auxin (AUX) content increased by 55% during the period of AR induction, but subsequently decreased by 23% during the remaining stages (Figure 2A). Cytokinin-zeatin riboside (CTK) content increased from S1 to S3, and then decreased during the final period of AR extension (Figure 2A). Gibberellic acid 3 (GA) content has a highest content at S2 (Figure 2A). Abscisic acid (ABA) increased by 25% during the AR induction stage, and no further significant differences were evident relative to S2, during the phases of AR initiation and extension (Figure 2A). Brassinolide (BR) content increased from S1 to S2, and then decreased by 28% from S3 to S4, but did not exhibit any significant differences between S2 and S3 (Figure 2A). Jasmonic acid (JA) content increased by 57% during the AR initiation phase, and then decreased by 34% during the AR extension phase (Figure 2A). In addition, the content of AUX in the IBA-treatment was significantly higher than what was observed in the control at S2 (Figure 2A). CTK content was significantly higher in the IBA-treatment than in the control at S3. In general, as compared with the control, treatment with IBA resulted in significant changes in the content of various hormones.

The ratios of AUX, CTK, GA, and ABA to each other were also determined over the time course of the experiment (Figure 2B). Compared with the control, the ratio of AUX/CTK in the IBA-treatment has significant differences at S2 and S3 (Figure 2B). The ratios of AUX/GA and ABA/AUX have significant differences at S2 between the IBA-treatment and the control (Figure 2B). No significant change in the ratio of GA/ABA, GA/CTK and ABA/CTK were observed between the IBA-treatment and the control (Figure 2B).

### 2.3. Expression Analysis of Related Genes with RNA Deep Sequencing and RT-qPCR

The samples that were sent for sequencing were only the IBA-treated ones. During the course of processing the Illumina sequencing reads, a total of 72,383 transcripts were obtained and 30,295 genes were detected as expressed. A total of 4294 genes exhibited significant differences in expression during AR formation. In order to determine the extent and depth of coverage for the level of sequencing, whether the selected reference genome was appropriate and to assess the reliability of the subsequent analysis; the proportion of mapped reads was compared and a number of descriptive statistics were generated (Table S2). Finally, a *p*-value < 0.05 was used to filter the data and approximately 446 genes among the annotated genes were selected based on their potential to be involved in AR formation (Tables S4–S8). An average total of 23,662,708, 26,570,345, 30,668,444, and 27,172,782 raw reads were obtained by Trimmomatic-0.32 software [26] for four stages (S1, S2, S3 and S4), respectively (Table S2). The overall alignment rates for the four stages were 63.22%, 64.21%, 65.08%, and 64.65%, respectively. The quality evaluation showed that the percentage of Q20 was >97% and the percentage of Q30 was >85%. The GC percentage in the four libraries ranged from 47% to 48% (Table S3). Overall, the quality values indicated that the sequence obtained by transcripts could be confidently used in the subsequent analyses.

**Figure 3:**
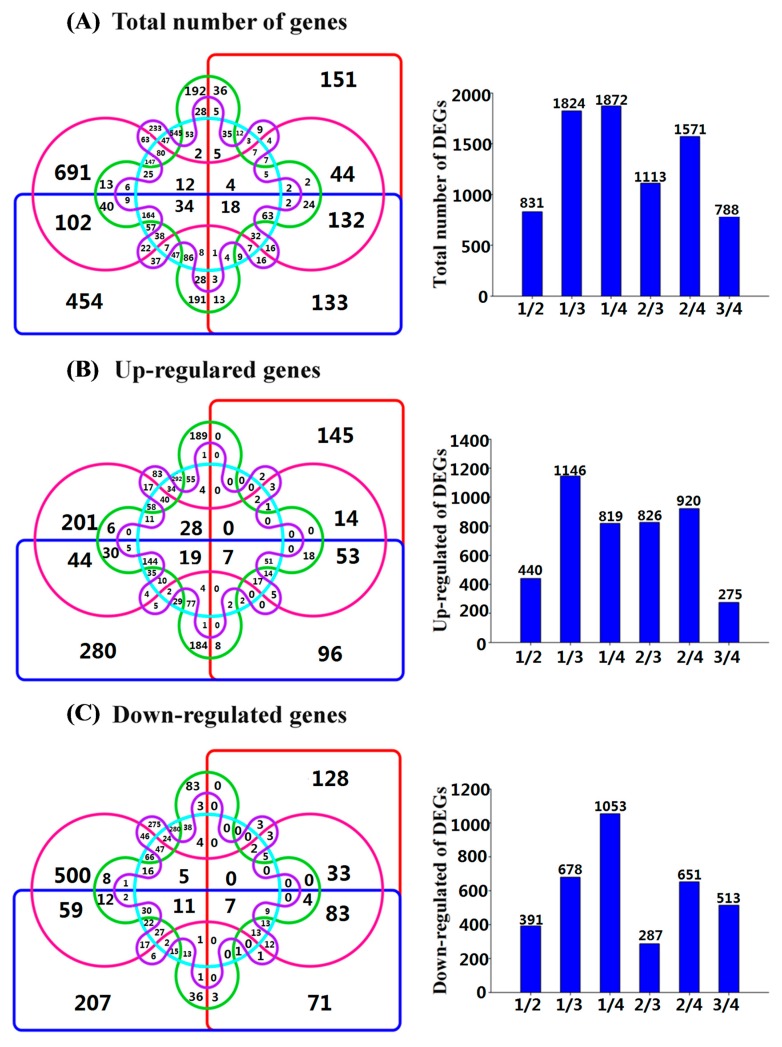
Venn diagrams of differentially expressed genes (DEGs) in stem cuttings of ‘T337’ apple root stocks during the different stages of AR formation. (**1/2**: S1 vs. S2, **1/3**: S1 vs. S3, **1/4**: S1 vs. S4, **2/3**: S2 vs. S3, **2/4**: S2 vs. S4, and **3/4**: S3 vs. S4 are the number of DEGs between S1 and S2, S1 and S2, S1 and S4, S2 and S3, S2 and S4, as well as S3 and S4). (**A**): Total number of DEGs; (**B**) up-regulated DEGs; (**C**) down-regulated DEGs. The bars indicate the number of total DEGs, up-regulated DEGs and down-regulated DEGs in S1 vs. S2, S1 vs. S3, S1 vs. S4, S2 vs. S3, S2 vs. S4, and S3 vs. S4, respectively.

In order to identify candidate genes that were specifically related to AR formation, a Venn diagram was conducted of the differentially expressed genes (DEGs) at the different stages of AR formation. Venn diagrams illustrating the DEGs in all the stages are presented in Figure 3 and the corresponding number of DEGs is illustrated in Figure 3. The total number of DEGs identified in S1 vs. S2, S1 vs. S3, S1 vs. S4, S2 vs. S3, S2 vs. S4, and S3 vs. S4 were 831, 1824, 1872, 1113, 1571 and 788, respectively (Figure 3A); in which the number of up-regulated genes were 440, 1146, 819, 826, 920, and 275, respectively (Figure 3B), and the number of down-regulated genes were 391, 678, 1053, 287, 651, and 513, respectively (Figure 3C).

The numbers of DEGs categorized into the three main GO categories (biological process, cellular component and molecular function) are presented in Figure S1. The screening of the DEGs was analyzed by GO enrichment and the topGO analysis software was used. Statistical significance was determined with the Fisher’s Exact Test. The GO analysis revealed that the number of DEGs involved in AR formation is associated with biological process, cellular component and molecular function. The GO analysis provided a hint on the experimental results, and found out that DEGs during the four stages of AR formation are related to which gene functions. A total of 122 KEGG (Kyoto Encyclopedia of Genes and Genomes) pathways were identified among the DEGs from the S1, S2, S3 and S4 profiles. The most heavily enriched KEGG pathways included metabolic, biosynthesis of secondary metabolites, plant hormone signal transduction, phenylpropanoid biosynthesis and phenylalanine metabolism pathways etc. In addition, hormones-, sugar-, cell cycle-, root development- and biological enzyme-related genes (Tables S4–S8) that were also selected from the heavily enriched KEGG pathways. A statistical analysis of the differentially expressed enrichment pathway diagram is presented in Figure S2. In addition, the expression of selected genes during AR induction, initiation and extension were identified by RT-qPCR. The gene name and primers are presented in Table S1.

Although the exact fold change of the DEGs at several data points varied between RNA-Seq and qPCR, the differential expression trends detected by the two approaches were largely consistent, indicating the reliability of the RNA-Seq results.

### 2.4. The Classification of Differentially Expressed Genes (DEGs)

The above-described DEGs and their corresponding RT-qPCR results were sorted and classified into seven categories. The results of the categories are as follows:

#### 2.4.1. Expression Profiles of Auxin-Related Genes

**Figure 4:**
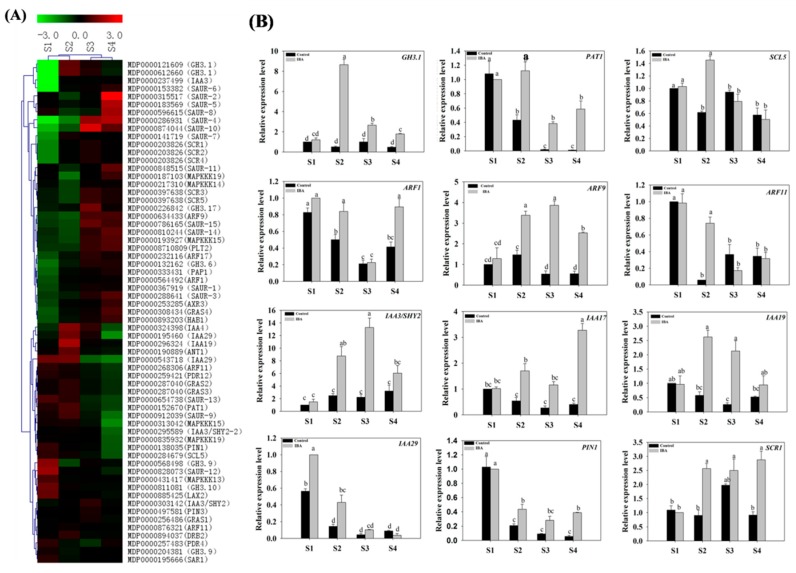
Expression profiles of auxin-related differentially expressed genes (DEGs) in stem cuttings of ‘T337’ apple root stocks during AR formation. (**A**) Heat map diagram of the log2 (FPKM) (Fragment Per Kilo bases per Million) values for genes annotated as auxin metabolism and transport pathway genes. Fold-change refers to the ratio of gene expression level in cuttings of ‘T337’ apple rootstocks; (**B**) expression of sugar-related genes as determined by RT-qPCR. Values represent means ± SE (*n* = 3). Different letters above the bars indicate a statistically significant difference.

Auxin-related genes, as well as key signaling components, were identified among the DEGs (Table S4). Representative genes included transcriptional regulator family genes (*IAA3/SHY2*, *AXR3/IAA17*, *IAA19*, *IAA29*); auxin efflux transport gene (*PIN1*); auxin response factors (*ARF1*, *ARF9*, *ARF11*); auxin-responsive gene family member (*GH3;1*); auxin polar transport-related gene (*PAT1*) and a GRAS family transcription factor (*SCR1*) (Figure 4A,B). A great majority of these genes exhibited a significant change in expression level between the different stages of AR development (S2 vs. S1, S3 vs. S1, S4 vs. S1, S3 vs. S2, S4 vs. S2, and S4 vs. S3). Although the exact fold change of the DEGs at several data points varied between RNA-Seq and qPCR, the differential expression trends detected by the two approaches were largely consistent (Figure 4).

Under IBA-treatment, the relative expression of *ARF9* (MDP0000634433) significantly increased from S1 to S3 (Figure 4B). In contrast, *ARF1* (MDP0000564492) and *ARF11* (MDP0000268306) were significantly down-regulated from S2 to S3 (Figure 4B). *PIN1* (MDP0000138035) was decreased by 57% from S1 to S2, but no significant difference was observed from S2 to S4 (Figure 4B). *IAA19* (MDP0000296324) was up-regulated during AR induction and then plateaued (Figure 4B). The relative expression of *IAA29* (MDP0000543718) exhibited a reduction from S1 to S2 but then decreased in expression by 91% from S2 to S4 (Figure 4B). *IAA17* (MDP0000253285) exhibited its highest expression level at S4 (Figure 4B). *IAA3/SHY2* (MDP0000303142) was up-regulated 7.8-fold from S1 to S3, but then decreased by 55% from S3 to S4 (Figure 4B). *GH3:1* (MDP0000121609) was significantly up-regulated from S1 to S2 and exhibited its highest expression level at S2 (Figure 4B). The relative expression of *PAT1* (MDP0000152670) increased by 12% from S1 to S2 and then decreased by 74% from S2 to S3 (Figure 4B). *SCR1* (MDP0000203826) was up-regulated 1.6-fold from S1 to S2 and then plateaued (Figure 4B). Collectively, RT-qPCR data indicated that the expression of representative auxin-related genes was significantly induced by exogenous IBA-treatment in relative comparison to the control (Figure 4B).

#### 2.4.2. Expression Profiles of CTK-Related Genes

**Figure 5:**
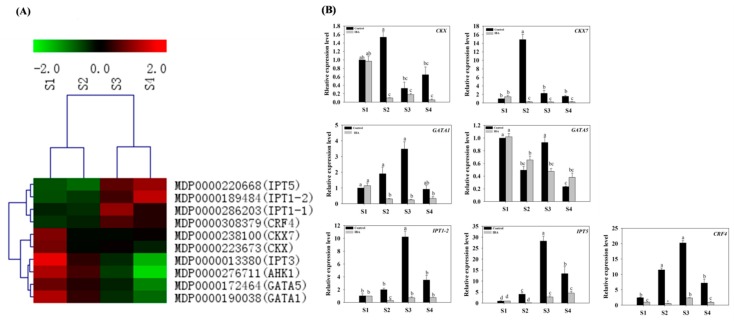
Expression profiles of cytokinin-related DEGs in stem cuttings of ‘T337’ apple rootstocks during AR formation. (**A**) Heat map diagram of log2 (FPKM) values in the expression of cytokinin-related genes. (**B**) Relative expression of cytokinin-related genes as determined by RT-qPCR. Different letters above the bars indicate a statistically significant difference. Values represent means ± SE (*n* = 3).

The expression pattern of CTK-related genes, selected from among the identified DEGs (Table S6), were examined. Representative genes included the CTK response factor 4 (*CRF4*), CTK oxidase gene (*CKX7*), histidine kinase 1 (*AHK1*), and the CTK-responsive gata factor 1 (*GATA1*), Isopentenyl adenosine transferase gene (*IPT*) etc. These genes were subjected to a clustering analysis (Figure 5A) and RT-qPCR was used to validate the expression of *CRF4* (MDP0000308379), *CKX7* (MDP0000238100), *GATA1* (MDP0000190038), *GATA5* (MDP0000172464), *IPT1-2* (MDP0000189484), and *IPT5* (MDP0000220668) during AR formation (Figure 5B). Results from the RT-qPCR analysis indicated that the expression patterns of the above-mentioned genes, in response to IBA-treatment, were largely consistent with the data obtained from RNA-seq data (Figure 5).

Under IBA-treatment, the relative expression of *CRF4* (MDP0000308379) increased 2.4-fold from S2 to S3, while at S1, S2, and S4 were similar to each other. *CKX7* (MDP0000238100) was down-regulated from S1 to S2 and then plateaued. Interestingly, *CKX* (MDP0000223673), *GATA1* (MDP0000190038) and *GATA5* (MDP0000172464) exhibited similar expression profiles with *CKX7* (MDP0000238100). Specifically, their highest levels of expression were detected at S1 and subsequently decreased by 55–75% from S1 to S4. *IPT1-2* (MDP0000189484) decreased by 70% from S1 to S2 and then increased approximately 1.6-fold from S2 to S4. *IPT5* did not exhibit any difference between S1 and S2, and then increased from S2 to S4. Consistent with the data pertaining to auxin-related genes, the CTK-related genes also exhibited a variable pattern of expression during AR formation. Collectively, the RT-qPCR results indicated that the expression patterns of representative CTK-related genes were significantly affected by exogenous IBA-treatment in relative comparison to the control (Figure 5B).

#### 2.4.3. Expression Profiles of GA-Related Genes

**Figure 6:**
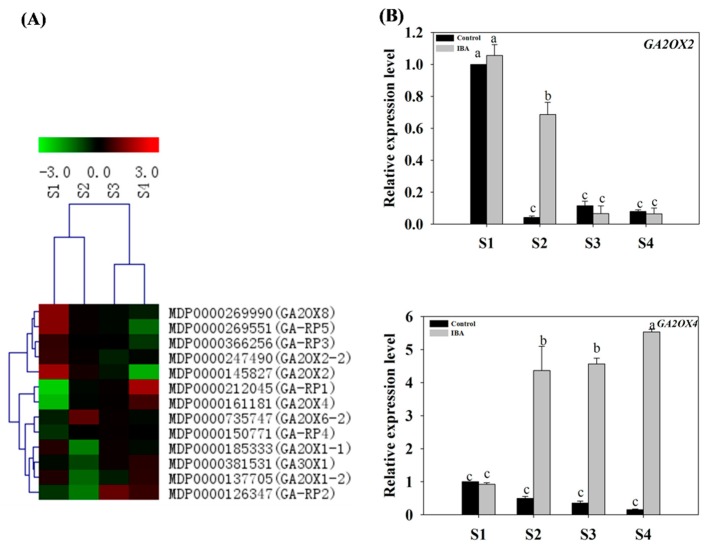
Expression profiles of GA-related DEGs in stem cuttings of ‘T337’ apple rootstocks during AR formation. (**A**) Heat map diagram of log2 (FPKM) values for genes annotated as gibberellic metabolism and signaling pathway genes. (**B**) Relative expression of GA-related genes as determined by RT-qPCR. Different letters above the bars indicate a statistically significant difference. Values represent means ± SE (*n* = 3).

The expression patterns of GA-related genes were selected from the DEGs database that were examined during AR formation. These genes included gibberellin 2-oxidases (*GA2OX1*, *GA2OX2*, *GA2OX6* and *GA2OX8*), gibberellins 3-oxidase 1 (*GA3OX1*), and gibberellin-regulated family protein genes (Table S6). In response to IBA-treatment, RT-qPCR analyses indicated that the expression trends of *GA2OX2* (MDP0000145827) and *GA2OX4* (MDP0000161181) were largely consistent with the RNA-seq data (Figure 6).

Under IBA-treatment, the relative expression level of *GA2OX2* was highest at S1 and lowest at S4 (Figure 6B). The expression profile of *GA2OX4* (MDP0000161181) was different from *GA2OX2* (*MDP0000145827*). The lowest expression value for *GA2OX4* was observed at S1 and then increased 3.7-fold from S1 to S2 and then plateaued (Figure 6B). Collectively, the RT-qPCR results indicated that the expression patterns of *GA2OX2* (MDP0000145827) and *GA2OX4* (MDP0000161181) were significantly affected by the exogenous IBA-treatment in comparison to the control (Figure 6B).

#### 2.4.4. Expression Profiles of Eth-, JA-, and BR-Related Genes

**Figure 7:**
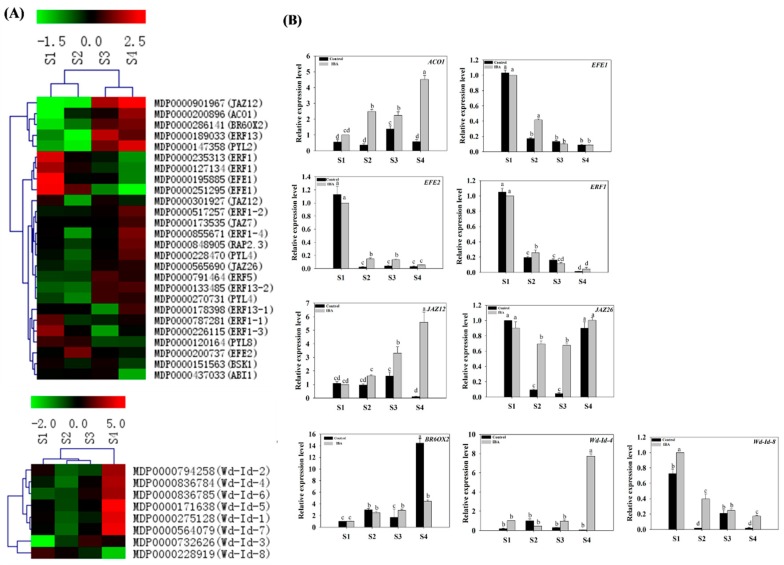
Expression profiles of ethylene-, brassinolide-, jasmonic acid-, abscisic acid-, and wound induction-related genes in stem cuttings of ‘T337’ apple rootstocks during AR formation. (**A**) Heat map diagram of log2 (FPKM) values for genes annotated as ethylene, brassinolide, jasmonic acid, abscisic acid and wound induction metabolism and signaling pathway genes. (**B**) Relative expression of ethylene-, brassinolide-, jasmonic acid-, abscisic acid-, and wound-induction-related genes as determined by RT-qPCR. Different letters above the bars indicate a statistically significant difference. Values represent means ± SE (*n* = 3).

Eth-, JA-, and BR-related genes were selected from the identified DEGs. These genes included ethylene-related genes included ACC oxidase 1 (*ACO1*), ethylene responsive element binding factors (*ERF1*, *ERF5* and *ERF13*), and ethylene-forming enzyme genes (*EFE1* and *EFE2*); JA-related genes included the jasmonate-zim-domain protein genes (*JAZ26*, *JAZ7*, and *JAZ12*); BR-related genes included BR-signaling kinase (*BSK1*), and brassinosteroid-6-oxidase 2 (*BR6OX2*) (Table S6). The expression levels of these genes were subjected to a clustering analysis (Figure 7A). Under treatment with IBA, although the DEGs at several data points varied between RNA-Seq and qPCR, the differential expression trends detected by the two approaches were largely consistent (Figure 7).

Under IBA-treatment, the relative expression level of *ACO1* (MDP0000200896) increased 1.5-fold from S1 to S2, and then increased 1-fold from S3 to S4. No significant differences were observed between S2 and S3 (Figure 7B). The expression of *EFE1* (MDP0000251295) was highest at S1, after which it was down-regulated by 89.9% from S1 to S3. No further changes in expression were observed between S3 and S4 (Figure 7B). The expression of *EFE2* (MDP0000200737) was significantly up-regulated from S1 to S2, and then down-regulated during the extension phase; with no further significant differences observed during the initiation phase (Figure 7B). The relative expression of *ERF1* (MDP0000235313) was significantly decreased during the AR induction phase, and then decreased further during the initiation and extension phases of AR formation (Figure 7B). No differences in the expression of *JAZ12* (MDP0000901967) were observed between S1 and S2; but then increased by 1.68-fold from S2 to S4 (Figure 7B). The relative expression level of *JAZ26* (MDP0000565690) decreased by 23% from S1 to S2, and then increased by 48% during the extension phases of AR formation (Figure 7B). The relative expression level of *BR6OX2* (MDP0000286141) increased 1.5-fold from S1 to S2 and then plateaued (Figure 7B). Collectively, the RT-qPCR results indicated that the expression level of representative Eth-, JA-, and BR-related genes were significantly affected by exogenous IBA-treatment in relative comparison to the control (Figure 7B).

#### 2.4.5. Expression Profiles of Wound Induction-Related Genes

Wound-induced-related genes were selected from among the DEGs and their expression profiles were examined during the four stages of AR formation. The selected genes included members of the wound responsive family genes (*Wd-Id-1*, *Wd-Id-2*, *Wd-Id-3*, *Wd-Id-4*, *Wd-Id-5*, *Wd-Id-6*, *Wd-Id-7* and *Wd-Id-8*) (Table S6). The expression levels of the selected genes in the four stages were subjected to a clustering analysis (Figure 7A). RT-qPCR was used to validate the expression of *Wd-Id-4* and *Wd-Id-8* that was derived from the RNA-seq data. Under the IBA-treatment, the expression trends of *Wd-Id-4* (MDP0000836784) and *Wd-Id-8* (MDP0000228919) showed largely similar trends between transcriptomics and RT-qPCR (Figure 7).

Under IBA-treatment, the relative expression level of *Wd-Id-4* (MDP0000836784) did not exhibit any significant changes during AR induction and initiation phases but then increased 7-fold during the extension phase of AR formation (Figure 7B). The relative expression of *Wd-Id-8* (MDP0000228919) decreased by 60% and 38% during AR induction and initiation phases, however, no significant changes in expression were observed during the extension phase (Figure 7B). Collectively, the RT-qPCR results indicated that the expression level of *Wd-Id-4* and *Wd-Id-8* were significantly affected by the exogenous IBA-treatment in comparison to the control (Figure 7B).

#### 2.4.6. Expression Profiles of Sugar Metabolism-Related Genes

**Figure 8:**
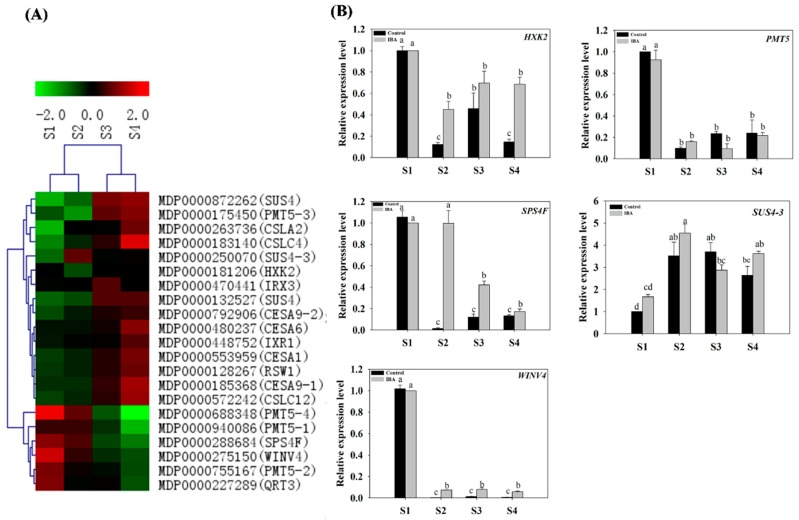
Expression profiles of sugar-related genes in stem cuttings of ‘T337’ apple rootstocks during AR formation. (**A**) Heat map diagram of log2 (FPKM) values for genes annotated as sugar metabolism and signaling pathway genes. (**B**) Relative expression of sugar-related genes as determined by RT-qPCR. Different letters above the bars indicate a statistically significant difference. Values represent means ± SE (*n* = 3).

Genes related to carbohydrate biosynthesis, as well as metabolic and sugar transport, were selected from the DEGs identified during the four stages of AR formation (Table S7) and subjected to a clustering analysis (Figure 8A). These genes included a syntheses 4 (*SUS4-1*, *SUS4-2* and *SUS4-3*), sucrose-phosphate synthase family protein gene (*SPS4F*), cellulose synthase (*CSLA2* and *CSLC4*), hexokinase 2 (*HXK2*), polyol/monosaccharide transporter 5 (*PMT5-1*, *PMT5-2*, *PMT5-3*, *PMT5-4*), cellulose synthase (*CESA1*, *CESA6*, *RSW1*, *CESA9-1*, *CESA9-2*), cellulose synthase family protein gene (*IRX1* and *IRX3*), cellulose-synthase-like (*CSLC4* and *CSLC12*) and a pectinlyase-like superfamily protein gene (*QRT3*). The expression patterns of several of the selected genes were also validated by RT-qPCR. Under the IBA-treatment, most of the expression profiles for the above-mentioned genes that were derived from the RNA-seq data were similar to the profiles obtained by RT-qPCR (Figure 8).

Under the IBA-treatment, the relative expression level of *HXK2* (MDP0000181206) and *PMT5* (MDP0000688348) decreased from S1 to S2, but no significant differences were observed from S2 to S4 (Figure 8B). The expression of *SUS4-3* (MDP0000250070) was up-regulated 1.7-fold from S1 to S2 and no further changes were observed during subsequent stages (Figure 8B). The relative expression of *SPS4F* (MDP0000288684) was similar at S1 and S2, but then decreased by 83% from S2 to S4 (Figure 8B). The relative expression of *WINV4* (MDP0000275150) decreased by 93% from S1 to S2 and then plateaued (Figure 8B). Collectively, the RT-qPCR results indicated that the relative expression levels of sugar-related genes were significantly affected by the exogenous IBA-treatment in comparison to the control (Figure 8B).

#### 2.4.7. Expression of Genes Related to Root Development, the Cell Cycle, and Proteinase Activity

**Figure 9:**
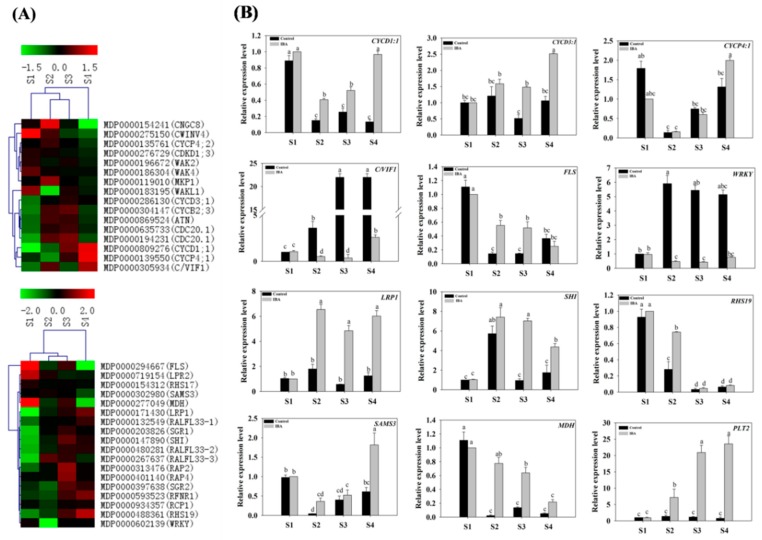
Expression profiles of root development-, cell cycle-, and enzyme- related genes in stem cuttings of ‘T337’ apple rootstocks during AR formation. (**A**) Heat map diagram of log2 (FPKM) values for genes annotated as root development-, cell cycle-, and enzyme-related genes. (**B**) Relative expression of root development-, cell cycle-, and enzyme-related genes as determined by RT-qPCR. Different letters above the bars indicate a statistically significant difference. Values represent means ± SE (*n* = 3).

Several root development-, cell cycle- and protease-related genes were selected from the identified DEGs (Table S5) and subjected to a clustering analysis (Figure 9A). These DEGs included a lateral root primordium protein-related (*LRP1*) gene, root hair specific 19 (*RHS19*), cell cycle-related genes (*CYCD1;1*, *CYCD3;1*, *CYCP4;1*), cell wall/vacuolar inhibitor of fructosidase 1 (*C/VIF1*) gene, WRKY family transcription factor (*WRKY*), peroxisomal NAD-malate dehydrogenase (*MDH*), Integrase-type DNA-binding superfamily protein gene (*PLT2*), S-adenosylmethionine synthetase family protein (*SAMS3*) gene, and a flavonol synthase1 (*FLS1*). The expression pattern of several of the selected genes (*LRP*, *RHS19*, *CYCD1:1*, *CYCD3:1* and *CYCP4:1*) were also validated by RT-qPCR. Under the IBA-treatment, although the exact fold change of the DEGs at several data points varied, the expression trends were largely similar between transcriptomics and RT-qPCR (Figure 9).

The Expression Profiles of Root Development-Related Genes

Under the IBA-treatment, the relative expression of *LRP1* (MDP0000171430) increased 5.5-fold from S1 to S2, and then plateaued (Figure 9B). The relative expression pattern of *RHS19* (MDP0000488361) was similar to *LRP1* (MDP0000171430). In addition, the relative expression of *SHI* (MDP0000147890) increased 8.2-fold during the induction phase and 12-fold during the initiation phase; followed by a 29% decrease during the extension phase of AR formation (Figure 9B).

The Expression Profiles of Cell-Cycle-Related Genes

Under IBA-treatment, the relative expression of *C/VIF1* (MDP0000305934), *CYCD1:1* (MDP0000809276), *CYCD3:1* (MDP0000286130), and *CYCP4:1* (MDP0000139550) exhibited their highest level of expression at S4. The relative expression of *C/VIF1* (MDP0000305934) was down-regulated by 51% from S1 to S2 and then increased 6-fold from S3 to S4 (Figure 9B). The relative expression of *CYCD1:1* (MDP0000809276) was also significantly up-regulated during the extension phase, but no further differences were observed between S2 and S3 (Figure 9B). The relative expression of *CYCD3:1* (MDP0000286130) increased by 69.7% from S3 to S4, but no further changes were observed from S1 to S3 (Figure 9B). However, the relative expression of *CYCP4:1* (MDP0000139550) decreased by 84% during the induction phase and then increased 2.3-fold during extension phase; no further changes were observed between S2 and S3 (Figure 9B). This pattern of expression was verified by RT-qPCR and added further confirmation that the expression of cell-cycle-related genes significantly increased during the extension stage of AR formation.

The Expression Profiles of Protease-Related Genes

Several protease-related genes were also identified from DEGs (Table S8) and were selected for further analysis (Figure 9B). The expression patterns of several of these genes were further validated by RT-qPCR. Under the IBA-treatment, the relative expression of *MDH* (MDP0000277049) decreased during the induction phase, and was further down-regulated from S2 to S4 (Figure 9B). The relative expression of *SAMS3* (MDP0000302980) was unaffected during the initiation phase of AR formation but then increased 2.5-fold from S3 to S4 (Figure 9B). The relative expression of *WRKY* (MDP0000602139) decreased by 51% during the induction stage but then increased by 77% during the extension phase (Figure 9B). The relative expression of *FLS1* (MDP0000294667) was down-regulated during the induction phase and then plateaued (Figure 9B). Lastly, the relative expression of *PLT2* (MDP0000871080) significantly increased from S1 to S3, with no further changes observed during the extension stage of AR formation (Figure 9B). Collectively, the RT-qPCR results indicated that the expression of representative root development-, the cell cycle-, and proteinase-related genes were significantly influenced by the exogenous IBA-treatment in comparison to the control (Figure 9B).

## 3. Discussion

### 3.1. Morphological and Anatomical Aspects of AR Formation in ‘T337’ Apple Rootstock

AR formation is a complex process consisting of three major phases (induction, initiation, and AR extension) which can occur in both juvenile and mature plants [27]. In the present study, AR formation in tissue cultured stem cuttings of the ‘T337’ apple rootstock was examined. Electron microscopy was used to observe AR formation at select stages (0, 3, 9, and 16 day) of AR formation (Figure 1). AR primordia originated from tissue near the vascular cambium and secondary phloem parenchymatous tissue. Continuous cell division, elongation and differentiation within the callus tissue resulted in the formation of root primordia [28,29], callus tissue, and subsequently differentiated ARs [30]. Importantly, the root primordia appeared to have originated from within the callus tissue (Figure 1). These observations provided basic information pertaining to the process of AR formation in apple rootstocks.

### 3.2. Multiple Hormones Signaling Pathways Interact with Auxin Signaling to Mediate IBA-Induced AR Formation in ‘T337’ Apple Rootstocks

To further characterize the differential expression of genes related to AR formation, gene expression under exogenous IBA-treatment was examined and revealed the induction of genes related to hormones-, wounding- and sugar-signaling related pathways etc. Numerous authors [31,32] have previously confirmed that auxin promotes AR formation. Synthetic forms of auxin, principally IBA, the compound is stillmainly used for the induction of AR formation [32]. However, there are very few studies in apple which focus on the molecular mechanisms of AR formation. Therefore, in the present study, treatment with IBA was chosen to override the effects from endogenous auxin. Similar results and methods were previously reported in mango [33] and petunia [34]: Indicating that endogenous and exogenous auxin play key roles in regulating AR formation via similar signalling pathways to some extent.

The interaction of different hormone networks plays an important role in plant development [35]; and in this regard, also determines the induction, initiation, and extension phases of AR formation [5,36]. The exogenous application of hormones affects the IBA-dependent AR formation and signaling pathways triggered by plant hormones interaction with those triggered by IBA [37]. Although we cannot conclude that the differentially expressed hormone-related genes contributed to AR formation, it is reasonable to hypothesize that some phytohormones do affect the IBA-induced AR formation.

### 3.3. Auxin Is Involved in the Regulation of AR Formation

Among the plant hormones, auxin plays a major role in the regulation of callus formation and AR organogenesis [17,38]. The present study found that auxin is associated with several biological processes as reflected by the genes whose expression was directly impacted by exogenous IBA-treatment. Under IBA-treatment, our data indicate that the majority of genes associated with auxin signal transduction *IAA19* (MDP0000296324), *IAA29* (MDP0000543718), *IAA3/SHY2* (MDP0000303142), *ARF9* (MDP0000634433) and *GH3.1* (MDP0000121609) are change during the AR induction phase (Figure 4B). In addition, the expression of *PAT1* (MDP0000152670) significantly decreased during the initiation phase of AR formation (Figure 4B). Our data also supports the view that wound-induced AR formation in cuttings is dependent on *PAT1* and the early accumulation of IAA in the rooting zone [7,39,40]. Additionally, Auxin content increased during the induction phase. All the results indicate that auxin is involved in the regulation of AR formation, especially during the induction phase. In addition, it is noteworthy that *GH3* gene’s family is an important class of early auxin-response genes involved in the development of the hypocotyls and roots in *Arabidopsis thaliana*, but the role of this gene family in woody plants is poorly understood [41]. The current study reports on the up regulation of *GH3* genes during AR formation in apple stem cuttings, which to some extent contradicts previous findings for other species [42]. However, a previous study also showed that the transcript abundance of nearly all *OsGH3* genes is enhanced on auxin treatment, with the effect more pronounced on *OsGH3-1*, *-2*, and *-4* in rice [43]. Under exogenous IBA treatment, *GH3* genes in different species may different functions in regulating plant development. Taken as a whole, *GH3* had a complex function and sophisticated mechanism of regulation in woody plants. Therefore, the specific molecular mechanism of *GH3* genes which regulate AR formation in apple rootstock still need further research.

### 3.4. Cytokinin Is Involved in the Regulation of AR Formation

**Figure 10:**
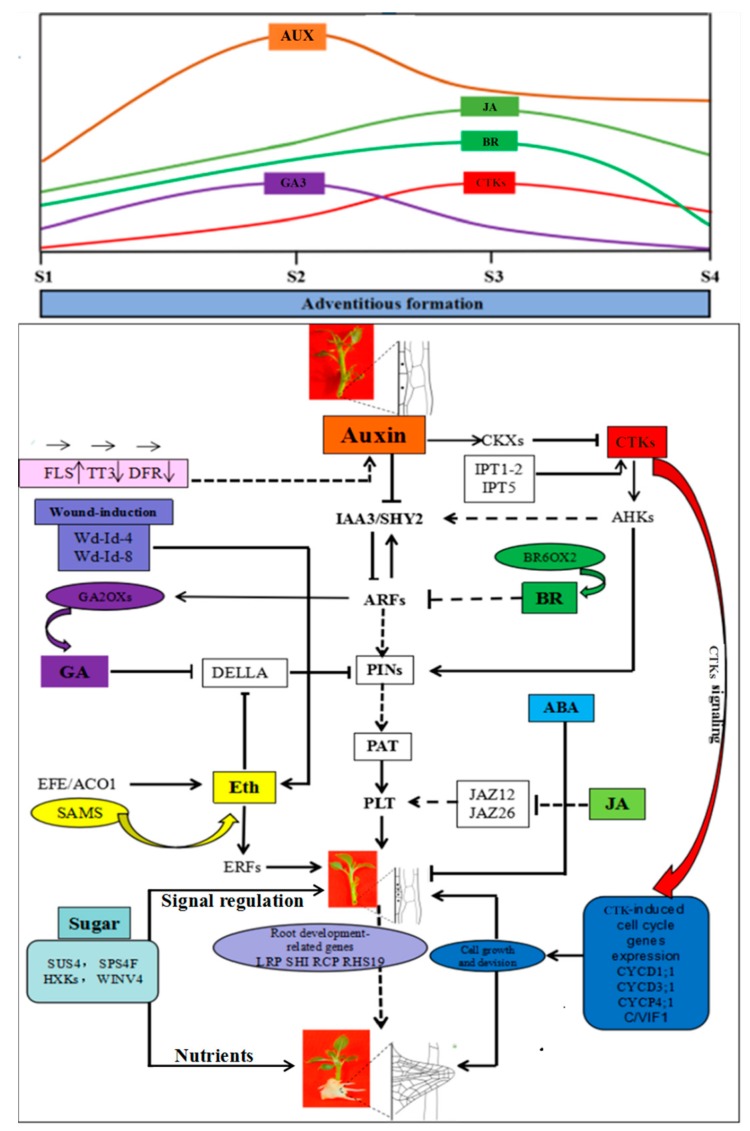
Hypothetical model for the regulation of adventitious root formation in stem cuttings of ‘T337’ apple rootstocks by hormones and sugar signaling crosstalk. AUX, Auxin; CTKs: Cytokinins; GA, Gibberellic acid; BR, Brassinolide; JA, Jasmonic acid; ABA, Abscisic acid; Eth, Ethylene; FLS, Flavonol synthase; TT3, DRF, Dihydroflavonol 4-reductase; IAA3/SHY2, AUX/IAA transcriptional regulator family protein; PINs, Auxin efflux carrier family proteins; PAT, auxin polar transport-related gene; PLT, Integrase-type DNA-binding superfamily protein gene; IPT1-2, Isopentenyl adenosine transferase gene 1-2; IPT5, Isopentenyl adenosine transferase gene 5; AHK, histidine kinases; CKXs, Cytokinin oxidases; BR6OX2, Brassinosteroid-6-oxidase 2; JAZ12, Jasmonate-zim-domain protein 12; JAZ26, Jasmonate-zim-domain protein 26; Wd-Id-4, Wound responsive family gene 4; Wd-Id-8, Wound responsive family gene 8; GA2OXs, Gibberellin 2-oxidases; DELLA, Gibberellic acid insensitive; EFE/ACO1, Ethylene forming enzyme; SAMS, S-adenosylmethionine synthetase; ERF, Ethylene response factor; SUS4, sucrose synthase 4; SPS4F, Sucrose-phosphate synthase family protein; HXKs, Hexokinases; WINV4, cell wall invertase 4; LRP, Lateral root primordium (LRP) protein-related; SHI, Lateral root primordium (LRP) protein-related; RCP, Root cap-related; CYCD1;1, CYCD3;1, CYCP4;1, Cell cycle-related genes; C/VIF1, Cell wall/vacuolar inhibitor of fructosidase 1. Different colored arrows indicate different types of hormone-related genes regulate AR formation in apple rootstock.

In contrast to AUX, CTK (Cytokinin) appears to play a role in AR formation [44]. CTK levels significantly increased during the induction and initiation phases of AR formation (Figure 2A). In accordance with previous studies, CTK appears to play a crucial role in regulating meristem activity during AR formation in apple rootstock stem cuttings [45]. Previous studies have demonstrated that a high ratio of AUX/CTK promotes AR formation [15]. CTK is antagonistic to auxin and suppresses rather than promotes AR formation in many other species; including *Arabidopsis*, rice, alfalfa (*Medicago sativa*), and poplar [10,45,46,47,48]. In addition, the effect of CTK on root formation was similar in wild-type and auxin mutants harboring auxin response- and transportation-associated defects [46]. In the current study, CTK levels increased during the induction phase and the ratio of AUX/CTK also increased during the AR induction phase, which may be due to the occurrence of cellular-programming and the need for cell division. In our study, CTK biosynthesis *IPT1-2* (MDP0000189484) and *IPT5* (MDP0000220668) exhibited high levels of expression but cytokinin oxidase genes *CKX* (MDP0000223673) and *CKX7* (MDP0000238100) exhibited lower expression during the extension phase (Figure 5). In addition, previous studies have also reported that CTK induces the expression of cell-cycle-related genes, and auxin-related genes, such as *SHY2* (MDP0000303142) and *PIN1* (MDP0000138035) [46,47,49]. These data show that the accumulation of CTK was promoted and highlight the importance of CTK during AR formation in apple rootstock (Figure 10). The increase in CTK levels may have been responsible for inducing the up-regulation of cell cycle-related genes to promote the division of stems cells and root hair formation. As determined by the AUX/CTK ratios, these data also confirm that alterations in CTK may function as feedback in regulating the expression of auxin-related genes such as *SHY2* and *PIN1* (Figure 10). Collectively, the present study indicates that CTK and AUX signaling pathways are partially independent during AR formation, their interaction is more than just a simple antagonism. It is plausible that a specific balance is most likely maintained between them in specific root regions. These proposed interactions, and the mechanisms underlying and associated with their interaction, remain to be further experimentally demonstrated.

### 3.5. Gibberellin Is Involved in the Regulation of AR Formation

There are relatively few studies on GA regulated root development in woody plants. Some previous reports showed that exogenous application of GA inhibits AR formation in rice plants, and rice mutants deficient in GA biosynthesis develop more ARs [50]. Likewise, the tomato pro (procera) mutant, in which GA signaling is constitutively active, has a very poor regenerative capacity when grown in a root-inducing medium [51]. In the present study, the expression profiles of GA-related genes induced by IBA-treatment may result in high levels of GA degradation and reduced levels of GA accumulation and then promote AR formation in apple rootstock. Additionally, previous studies have also demonstrated that GA homeostasis affects the expression of *PAT*, and thus changes the ability to accumulate auxin in different plant tissues [52]. GA promotes the proteasome-mediated degradation of DELLA proteins [53]. Therefore, the current study infers that GA, through DELLA, repressed the expression of *PINs*; and further resulted in decreased expression levels of *PAT* which consequently affected the accumulation of auxin (Figure 10). Based upon these data, these data support the hypothesis that GA-related genes may mediate the interaction of GA and auxin to control AR formation (Figure 10). However, the current study data indicated that auxin levels were not depressed by changes in the level of GA, as reflected by changes in the AUX/GA ratio during AR formation. The results indicated that GA can also affect auxin localization which may be more important than overall levels of auxin during AR formation. Our data indicated that auxin levels were not depressed by changes in the level of GA, as reflected by changes in the AUX/GA ratio during AR formation. In general, our findings suggest that the GA signaling pathway interacted with auxin signaling pathway to mediate AR formation. The results also help us better understand the mechanism of AR development in apple rootstock. However, the mechanisms underlying and associated with their interaction, remain to be further experimentally demonstrated.

### 3.6. Ethlene, Jasmonic Acid, and Brassinolide etc. Are Involved in the Regulation of AR Formation

Other hormones, including Eth, JA, and BR also appeared to affect AR formation. ARs often form directly from sites in cuttings that were wounded or indirectly from wounded vascular tissues [13,14,54]. Ethylene positively regulates AR formation in flooded tomato plants [55], and a promoting role of Eth in AR development has been reported in species such as sunflower (*Helianthus annuus*), apple, mung bean (*Vigna radiata*), and petunia (*Petunia* sp.) [56,57]. JA has been recently shown to be a negative regulator of adventitious rooting that acts downstream of the auxin pathway in *Arabidopsis* [20]. In the current study, JA had no significant difference between control and IBA-treatment in S1 and S2, but increased in S3. We speculated that JA may play a role in regulating the induction phase (from S1 to S2) of adventitious root development in apple rootstock and its function in S3 may not be strong. The current study provided a basis for the study of JA-mediated AR formation in apple rootstock. However, the specific mechanisms of regulating JA response in apple rootstock still need in-depth research.

Similar to auxin, BR promotes primary root growth at low concentrations but inhibits it at higher concentrations [58]. These hormones appear to regulate LR (lateral root) development through a complex interplay with auxin [59,60,61]; whether these hormones also interact with auxin during AR formation in apple rootstock is not clear. In this study, treatment with an exogenous application of IBA promotes AR formation and RT-qPCR results indicated that the expression of DEGs is also affected by IBA-treatment as compared to the control (Figure 9B). Therefore, this study concluded that changes in the expression of Eth-, JA-, and BR-related genes *ACO1* (MDP0000200896), *EFE1* (MDP0000251295), *EFE2* (MDP0000200737), *ERF1* (MDP0000235313), *JAZ12* (MDP0000901967), *JAZ26* (MDP0000565690), and *BR6OX2* (MDP0000286141) were associated with AR formation (Figure 9B). The current results suggest that these hormones may interact with auxin to mediate AR formation. Although the specific interactions between these hormones and hormone-associated genes have not been experimentally verified in apple rootstocks, their involvement in AR formation is supported by previous studies in other species [4,15,55,58,59]. Collectively, these results serve as a foundation to provide a direction of AR development in future experimental strategies.

### 3.7. Wound Signaling and AR Formation in ‘T337’ Apple Rootstocks

AR formation arises from wounded stem cuttings [54]. Ethylene is synthesized as a stress response signal to wounding [62], and thus plays a prominent role in AR formation [56,57]. Wound-induced genes can serve as metabolic signals, and as a result, regulate tissue hormone levels (Table S7, Figure 2A). Our results indicate that several wound-induced genes, such as *Wd-Id-4* (MDP0000836784) and *Wd-Id-8* (MDP0000228919), are involved in regulating AR formation (Figure 7B). Experimental data support the view that wound-induced AR formation in cuttings is dependent on *PAT1* and involves an early accumulation of IAA in the rooting zone [7,39,40]. Therefore, we hypothesize that a wounding signal first induces ethylene production and then AR formation is subsequently promoted by an accumulation of auxin (Figure 10): Which represents a relatively new point of view on the interaction of wounding signal and ethylene pathways-mediated AR development in apple rootstocks. However, additional in-depth research is warranted and necessary to completely elucidate the specific mechanisms regulating this response.

### 3.8. Sugar Signaling Mediated AR Formation in ‘T337’ Apple Rootstock

Sugars serve as both prime energy sources and also as signaling molecules that control plant growth and development [63]. The role of sucrose in dormancy development, storage organ formation and maturation of somatic embryos has been previously examined [64,65]. In apple microcuttings, the type of sugar is known to influence root regeneration and the concentration of sucrose affects the number of ARs [66]. In addition, an interaction was observed between sucrose and auxin which functioned to mediate AR formation [67]. Analyses of the DEGs related to sugar biosynthesis genes in ‘T337’ apple rootstock were consistent with previous studies. For example, the relative expression levels of *SUS4-3* (MDP0000250070) and *SPS4F* (MDP0000288684) were high during the induction stage (Figure 8B and Table S7). Sucrose synthase (*SUS*) cleaves sucrose in a reversible reaction, producing UDP-glucose and fructose [68,69]. *SUS* activity has been related to sink strength determination and storage functions [70]. Under IBA-treatment, the present study determined that the expression profiles of *SUS4-3* (MDP0000250070) and *SPS4F* (MDP0000288684), which would result in the localization of high level of soluble sugar, were high during the induction phase of AR formation. This finding was in accordance with reports showing that soluble sugars levels play an important role during the induction phase of AR and that fructose content is closely related to rooting ability [71]. The expression level of *PMT5* (MDP0000688348) was high during the induction phase and was ultra-low during the extension phase; indicating that *PMT5* may also play an important role in regulating AR formation. *HXK* has been identified as a glucose sensor, independent of its enzymatic role in converting glucose to glucose 6-phosphate in *Arabidopsis* [72,73]. Therefore, the observed pattern of *HXK2* (MDP0000181206) expression may have resulted from the accumulation of soluble sugars during the induction phase of AR formation; which may have induced the up-regulation of *HXK2* expression in order to increase the sugar signal response sensitivity. *HXK2* regulates the transcription of glucose-related genes in a glucose-dependent manner, and also participates in hormone signaling. In poplar, *WINV4* plays a key role in sucrose metabolism in various tissues and organs [74]. Our data indicated that *WINV4* (MDP0000275150) also played a role in the induction phase of AR formation (Figure 8B). Changes in the expression of glucose-related genes were observed by IBA-treatment. The results indicated that sugar, via the activity of *SUS4-3* (MDP0000250070), *SPS4F* (MDP0000288684), *HXK2* (MDP0000181206), and *WINV4* (MDP0000275150), is likely transported into the basal portion of the rootstock stems undergoing AR formation; thus providing sufficient energy and signal activity required for adventitious rooting to occur (Figure 10).

### 3.9. Cell Cycle- and Root Development-Related Gene Expression during AR Formation

Active cell division and differentiation primarily occurs in the root meristem zone during AR formation and the extent of this activity may regulate the rate of root growth and development [5,75,76]. The stimulatory effect of auxin on cell division is strongly linked to cell cycle processes. In fact, many cell cycle-related genes are up-regulated in roots by auxin [77,78], however, CTK regulated cell division is also well established. Results of our analyses indicate that several cell-cycle-related genes, including *CYCD1:1* (MDP0000809276), *CYCD3:1* (MDP0000286130)*, CYCP4:1* (MDP0000139550), and *C/VIF1* (MDP0000305934) exhibited their highest expression level at S4 (Figure 9B and Table S5). These data suggest that these genes may be involved in promoting cell division in response to signals generated during AR formation (Figure 10). In addition, *C/VIF1* plays a key role in the regulation of cell wall development and changes in cell walls within the rooting zone can play an important role in regulating cell division and differentiation during AR formation [79]. In general, the stage-specific expression of the analyzed cell-cycle-related genes highlights their importance in the molecular mechanisms regulating AR formation.

Previous studies indicated that *LRP1* (*MDP0000171430*) is a marker gene for the induction stage of AR formation in Arabidopsis [80,81], and *RHS19* (*MDP0000488361*) is known to regulate root hair development [82]. As shown in the present study, the relative expression levels of *LRP1* and *SHI* were significantly higher during the extension phase than the induction phase of AR formation. Additionally, *RHS19* expression gradually increased by IBA-treatment in comparison to the control; especially at S2 (Figure 9B and Table S5). These data suggest that the aforementioned genes may be involved in the regulation of AR development; especially in the induction and extension in ‘T337’ apple rootstocks.

## 4. Material and Methods

### 4.1. Plant Material, Growth Conditions, and Treatments

Stem cuttings of ‘T337’ apple rootstock were cultivated in tissue culture at the Northwest Agriculture and Forestry University, Yangling, China. The tissue culture cuttings were maintained under a 16/8 h light/dark cycle at 25 ± 1 °C, followed by 8 h 15 ± 1°C. Under these conditions, the relative humidity was approximately 70~80%. The rootstock cuttings were treated with indole-3-butyric acid (IBA), which is widely used to promote adventitious rooting. Separate rootstock cuttings served as controls and were not treated with IBA. The cuttings were randomly divided into two groups; IBA treatment and control material. The cuttings of the IBA treatment group were transferred into a medium composed of 1/2 MS, 1 mg·L^−1^ IBA, 20 g·L^−1^ sugar and 8 g·L^−1^ agar, pH 5.8. The cuttings of the control group were treated with IBA-free medium. Samples were harvested at the four stages of AR formation based on morphological changes. Stage 1 (S1) represents stem cuttings treated with 1 mg·L^−1^ IBA at 0 day (0 day, competent cells); stage 2 (S2) represents stem cuttings in which AR formation was induced at 3 day (3 day, Cell cycle reactivation); stage 3 (S3) represents stem cuttings in which callus formed at the base of the stem at 9 day (9 day, Activation of AR primordium formation); and stage 4 (S4) represents stem cuttings in which AR broke through the epidermis and AR emerged at 16 day (16 day, AR outgrowth). Based on previous research, the S1 to S2 period of growth was considered as the AR induction phase, S2 to S3 was defined as the AR initiation phase, and the S3 to S4 stages were defined as the AR extension phase. The experimental errors were managed by using a complete randomized design for three biological replicates, each including at least 60 stem cuttings. Samples consisted of basal portions of the stems (approximately 0.5 cm) containing the AR zone. The collected samples were immediately immersed in liquid nitrogen and stored at −80 °C until used for further processing. Cuttings of IBA treated material (S1, S2, S3, S4) were used for RNA-seq with three biological replicates and 12 libraries were sequenced. Cuttings of both the IBA treatment and control were used for analysis of paraffin section, hormone levels and gene expression.

### 4.2. Paraffin Section

Samples collected for fixation, paraffin embedding and sectioning were processed using previously published protocols [83,84]. All samples were collected from three biological replicates and *n* = 10 in each replicate.

### 4.3. Extraction and Measurement of Hormones in Cuttings

Auxin (AUX), Cytokinin (zeatin riboside) (CTK), Gibberellic acid 3 (GA), Brassinolide (BR), Jasmonic acid (JA), and Abscisic acid (ABA) contents between stem cuttings harvested from IBA-treatment and control were determined using an enzyme linked immunosorbent assay (ELISA). [85,86]. Approximately 0.6 g samples were thorough grinded in liquid nitrogen, and then treated with cold 80% (*v*/*v*) methanol and 1 mM butylated hydroxytoluene overnight for about 12 h at 4 °C. In order to collect extracts, the mixture was centrifuged (10,000× *g* for 20 min at 4 °C), passed through a Sep-Pak C_18_ cartridge and dried under N_2_. The resulting residues were dissolved in phosphate buffer. The ELISAs used to analyze AUX, CTK, ABA, JA, BR and GA3 contents were completed in 96-well microtitration plates. The coated plates were incubated for 40 min at 37 °C after adding standard hormone, sample extracts and antibodies. After rinsing plates, 100 μL peroxidase-labeled goat antirabbit immunoglobulin was added to each well, and the plates were incubated for 40 min at 37 °C. Colored substrate (*O*-phenylenediamine) was added to each well, for enzymatic reaction at 37 °C for 15 min, and the reaction was halted by the addition of 3 M H_2_SO_4_. Absorbances at 490 nm were determined with an ELISA spectrophotometer and used to calculate AUX, CTK, ABA, JA, and GA3 concentrations. All antibodies against each hormone were monoclonal and were obtained from the Center of Plant Growth Regulator, China Agricultural University. Three biological replicates were used for each hormone analysis (200 mg per replicate).

### 4.4. RNA Extraction and cDNA Synthesis

Total RNA was extracted using a CTAB-based method [87], with slight modifications as follows. Samples were ground in liquid nitrogen with the aid of a mortar and pestle and added to 900 μL of extraction buffer (2% CTAB, 2.5% PVP-40, 2 M NaCl, 100 mM Tris–HCl, pH8.0, 25 mM EDTA, pH8.0, and 2% *β*-mercaptoethanol pre-heated to 65 °C just prior to use), inverted for 5 min, and then incubated at 65 °C for 5 min. The samples were then centrifuged at 12,000× *g* for 10 min at 4 °C. The supernatant was collected and extracted twice with an equal volume of chloroform/isoamyl alcohol (24:1 *v*/*v*). The RNA was precipitated with 3 M LiCl (final concentration) and resuspended in 500 μL of SSTE buffer (10 mM Tris–HCl, pH 8.0, 1 mM EDTA, pH 8.0, 1% SDS, 1 M NaCl) pre-heated to 65 °C. Then, an equal volume of chloroform/isoamyl alcohol was added to each tube which was subsequently centrifuged at 12,000× *g* for 10 min at 4 °C. The supernatant was collected and the RNA was precipitated with 2.5 volumes of ethanol at −80 °C for at least 30 min. Finally, the RNA was pelleted by centrifugation at 12,000× *g* for 20 min at 4 °C, washed with 70% ethanol, dried and resuspended in DEPC–water. The integrity of the total RNA was verified by running samples on 2% agarose gels. cDNA was synthesized using a PrimeScript RT Reagent Kit with gDNA Eraser (TaKaRa Bio, Shiga, Japan).

### 4.5. cDNA Library Construction and RNA Deep Sequencing

Total RNA was extracted from stem cutting samples that were collected at 0, 3, 9 and 16 days from IBA-treated cuttings. These extracts were then used for the construction cDNA libraries and subsequent sequencing. The library construction was performed according to a previously published protocol [88]. A total of 12 libraries were sequenced using an Illumina HiSeq TM2000 (BGI, Shenzhen, China) platform.

Statistical Analysis of Clean Reads

Paired-end DNA sequencing results contained low-quality reads and the end contained the unknown base N reads. In order to ensure the quality of downstream data generation and subsequent analyses on clean data, we filtered the next generation sequencing machine data using Trimmomatic (version 0.32) software [26]. For trimming slide windows, filtering was set to 4 when the average base was less than 15 bytes of the reads. The N bases were removed at both ends of the reads; preserving the truncated length longer than the 114nt reads. The clean data were filtered by Trimmomatic and FastQC software was subsequently used for its quality-related information statistics.

Mapping Rate Statistics

The sample reads were compared with the reference genome (MalDomGD1.0, ftp://ftp.ncbi.nlm.nih.gov/genomes/all/GCF_000148765.1_MalDomGD1.0/GCF_000148765.1_MalDomGD1.0_genomic.fna.gz) (Default parameter) using HISAT2 (version 2.0.1-beta) software [89].

The Distribution of Reads in The Reference Genome

Statistical analyses were performed on the various structural regions of reads, and compared on the genome. The positions of reads were divided into Exons (exon), Introns (intron) and transcription initiation sites; in proximity to 1 k, 5 k and 10 k regions near the termination site. Under normal circumstances, the Exon (exon) region of the sequencing positioning percentage should be the highest, and the other regions of the sequencing positioning percentage should be smaller than the Exon (exon) region.

Detection of New Transcription Sites

Stringtie software was used to assemble reads and cuffcompare enabled the comparison of the assembled results with known genes in the genomic annotation document. A total of 11,833 isolates were identified (the interval for the annotated gene is included in the annotation file of reference genome), the new predictive transcription site sequence was extracted and CPC software [90] predicted its coding ability.

Gene Expression Abundance Statistics

Gene expression levels are estimated by counting the reads that are localized to exon regions of the gene. The reads count is proportional to the true expression level of the gene and is positively correlated with gene length and sequencing depth. In order to compare expression levels between different genes and experiments, the concept of FPKM (Fragment Per Kilo bases per Million) reads was introduced. FPKM is the number of PEs per kilobase length per million reads from a gene. FPKM also considers the effect of sequencing depth and gene length on the counting of reads. The FPKM values can reflect the gene expression. In the current study, FPKM ≥ 1 was used as a standard for identifying gene expression. The weakly expressed genes were filtered out by this standard.

Differentially Expressed Genes (DEGs)

For differential expression analyses, the documented transcripts within the reference genomic annotation file, as well as the newly assembled transcripts from String Tie, were analyzed by ballgown (version 2.2.0) software [91]. Screening criteria for the differential expression isoforms were |log2FC| ≥ 1 and *p*-value < 0.05. Two strategies were used to analyze the DEGs. The first was gene ontology functional enrichment and the second was pathway enrichment. GO enrichment analysis of functional significance applied a Fisher’s Exact Test for mapping all DEGs to terms in the GO database. topGO (version 2.18.0) software was used for the GO enrichment analysis and screening of differentially expressed genes [92]. The *p*-value was corrected by the Bonferroni test and a corrected *p*-value < 0.05 was chosen as the threshold to define a significantly enriched GO term. This analysis allowed us to identify the major biological functions of the DEGs. The KEGG Pathway enrichment analysis identifies significantly enriched metabolic pathways or signal transduction pathways. KOBAS (kobas2.0-20150126) analysis software [93] was used for KEGG Pathway enrichment analysis and statistical analyses were performed with the Hypergeometric Test. Pathways with *p*-values < 0.05 were defined as significantly enriched. In addition, this study also combined previous research backgrounds to screen for DEGs.

### 4.6. RT-qPCR Analysis

In this study, the expression patterns of candidate genes were validated by RT-qPCR. Specifically, genes involved in hormone metabolism (AUX, CTK, GA, BR, JA, ABA, Eth), sugar, cell cycle, root development, and enzyme activity were analyzed. The identification of gene functions was based on reports that were derived from other species, and the sequences of the corresponding genes were used as the query in GenBank to identify the gene homologue and MDP number in apple. The sequences of the designed primer pairs were selected based on apple data (*Malus* × *domestica*) that were published in GenBank using Primer 6.0 (Genetyxv. 10) software. The sequences of the utilized gene-specific primers are listed in Table S1. The RT-qPCR assay mix consisted of 2 μL cDNA (diluted 1:16), 10 μL 2 × SYBR Premix ExTaq II (TaKaRa Bio), 0.8 μL primer pair (10 μM), and 6.4 μL ddH_2_O. The RT-qPCR assay was conducted on an iCycler iQ Real Time PCR Detection System (Bio-Rad, Xi’an, China) with an initial denaturation of 3 min at 95 °C, followed by 40 cycles of 15 s at 94 °C, 20 s at 60 °C and 20 s at 72 °C. An apple *ACTIN* gene was used for normalization. Each of the analyzed samples consisted of three biological and technical replicates. The 2^−ΔΔ*C*t^ method was used to calculate the relative expression of the analyzed genes [94].

### 4.7. Time-Series Cluster Analysis and Hierarchical Clustering

The time-series cluster method was used to analyze the expression profiles of the DEGs [95] and the raw abundance values were converted to FPKM. Subsequently, unique profiles were defined using a strategy for clustering times-series gene expression data during four sampling time points (S1, S2, S3 and S4). The expression model profiles were related to the actual or the expected number of genes assigned to each profile’s model. Using the Fisher’s exact and multiple comparison tests, significant profiles had higher probabilities than expected. In addition, hierarchical clustering heat maps were generated using Multi Experiment Viewer software 4.2 (MEV4.2) (http://www.tm4.org/mev.html) and the log_2_ (FPKM) values of each gene. The Pearson correlation with complete centroid linkage was adopted for all clustering analyses.

### 4.8. Statistical Analysis

Statistical processing of plant phenotype data, hormone content, and RT-qPCR data was performed in Excel 2007. Differences among means were evaluated with the Statistical Program for Social Science 19 (SPSS, Chicago, IL, USA) using a two-tailed *t*-test at the 5% level. Graphs were generated in Excel 2007 and SigmaPlot10.0 (Systat Software, Inc., Chicago, IL, USA).

## 5. Conclusions

The current study proposes a preliminary model which describes the important biological processes occurring during AR formation in apple rootstocks. AR formation involves the regulation of hormones (including hormones biosynthesis, conjugation, transport, and degradation), carbohydrate metabolism, and the regulation of cell-cycle-related genes and suites of genes related to AR differentiation. Many sugar-, wounding-, root development-, and cell cycle-related genes interact with auxin signaling pathway-related genes to ultimately control AR formation (Figure 10). Our study provides a foundation to enable further characterization to better understand the association of candidate genes to various pathways that regulate AR formation. While the current study provides an overview of the transcriptomic changes that occur during AR formation in ‘T337’ apple rootstocks, the specific interactions and mechanisms, though proposed, still need to be experimentally demonstrated.

AR formation is a complex process involving various regulatory pathways. Our work provided a transcriptomic overview pertaining to IBA-induced AR formation. However, owing to technical limitations, our study did not provide any post-transcriptional or (post)-translational evidence for AR formation, which are also important regulatory pathways for AR formation. Whether these pathways are also involved in the IBA-induced AR formation still needs further analysis.

## Supplementary Materials

The following are available online at http://www.mdpi.com/1422-0067/19/8/2201/s1.

## Abbreviations

AR	Adventitious root
LR	Lateral root
IBA	Indole-3-butyric acid
IAA	Indole-3-acetic acid
ELISA	enzyme linked immunosorbent assay
PI	primordium initiation.
RP	root primordium
KEGG	Kyoto Encyclopedia of Genes and Genomes
FPKM	Fragment Per Kilo bases per Million
DEGs	Differentially Expressed Genes
AUX	Auxin
CTK	Cytokinin
GA	Gibberellic acid
Eth	Ethylene
BR	Brassinolide
JA	Jasmonic acid
ABA	Abscisic acid

## References

[1] Li S.W., Shi R.F., Leng Y. (2015). De Novo Characterization of the Mung Bean Transcriptome and Transcriptomic Analysis of Adventitious Rooting in Seedlings Using RNA-Seq. PLoS ONE.

[2] Bellini C., Pacurar D.I., Perrone I. (2014). Adventitious Roots and Lateral Roots: Similarities and Differences. Annu. Rev. Plant Biol..

[3] Ludwig-Müller J., Vertocnik A., Town C.D. (2005). Analysis of indole-3-butyric acid-induced adventitious root formation on Arabidopsis stem segments. J. Exp. Bot..

[4] Della R.F., Fattorini L., D’Angeli S., Veloccia A., Falasca G., Altamura M.M. (2013). Auxin and cytokinin control formation of the quiescent centre in the adventitious root apex of Arabidopsis. Ann. Bot..

[5] Atkinson J.A., Rasmussen A., Traini R., Voss U., Sturrock C., Mooney S.J., Wells D.M., Bennett M.J. (2014). Branching out in roots: Uncovering form, function, and regulation. Plant Physiol..

[6] Ahkami A.H., Melzer M., Ghaffari M.R., Pollmann S., Ghorbani J.M., Shahinnia F., Hajirezaei M.R., Druege U. (2013). Distribution of indole-3-acetic acid in Petunia hybrida shoot tip cuttings and relationship between auxin transport, carbohydrate metabolism and adventitious root formation. Planta.

[7] Garrido G., Ramon G.J., Angel C.E., Acosta M., Sanchez-Bravo J. (2002). Origin and basipetal transport of the IAA responsible for rooting of carnation cuttings. Physiol. Plant.

[8] Da C.C., de Almeida M.R., Ruedell C.M., Schwambach J., Maraschin F.S., Fett-Neto A.G. (2013). When stress and development go hand in hand: Main hormonal controls of adventitious rooting in cuttings. Front. Plant Sci..

[9] Klerk G.J.D., Guan H., Huisman P., Marinova S. (2011). Effects of phenolic compounds on adventitious root formation and oxidative decarboxylation of applied indoleacetic acid in Malus ‘Jork 9’. Plant Growth Regul..

[10] Ramirez-Carvajal G.A., Morse A.M., Dervinis C., Davis J.M. (2009). The cytokinin type-B response regulator PtRR13 is a negative regulator of adventitious root development in Populus. Plant Physiol..

[11] Busov V., Meilan R., Pearce D.W., Rood S.B., Ma C., Tschaplinski T.J., Strauss S.H. (2006). Transgenic modification of gai or rgl1 causes dwarfing and alters gibberellins, root growth, and metabolite profiles in Populus. Planta.

[12] Bergonci T., Ribeiro B., Ceciliato P.H., Guerrero-Abad J.C., Silva-Filho M.C., Moura D.S. (2014). Arabidopsis thaliana RALF1 opposes brassinosteroid effects on root cell elongation and lateral root formation. J. Exp. Bot..

[13] Chen C.W., Yang Y.W., Lur H.S., Tsai Y.G., Chang M.C. (2006). A novel function of abscisic acid in the regulation of rice (*Oryza sativa* L.) root growth and development. Plant Cell Physiol..

[14] Negi S., Sukumar P., Liu X., Cohen J.D., Muday G.K. (2010). Genetic dissection of the role of ethylene in regulating auxin-dependent lateral and adventitious root formation in tomato. Plant J..

[15] Zhang J., Zhang X., Wang Y., Lin M. (2014). The Dynamic Changes of Endogenous Hormones in the Process of Adventitious Root Formation for Seabuckthorn Cutting Seedling. Glob. Seabuckthorn Res. Dev..

[16] Chen L., Tong J., Xiao L., Ruan Y., Liu J., Zeng M., Huang H., Wang J.W., Xu L. (2016). YUCCA-mediated auxin biogenesis is required for cell fate transition occurring during de novo root organogenesis in Arabidopsis. J. Exp. Bot..

[17] Lavenus J., Goh T., Roberts I., Guyomarc’H S., Lucas M., De Smet I., Fukaki H., Beeckman T., Bennett M., Laplaze L. (2013). Lateral root development in Arabidopsis: Fifty shades of auxin. Trends Plant Sci..

[18] Legue V., Rigal A., Bhalerao R.P. (2014). Adventitious root formation in tree species: Involvement of transcription factors. Physiol. Plant.

[19] Gutierrez L., Bussell J.D., Pacurar D.I., Schwambach J., Pacurar M., Bellini C. (2009). Phenotypic plasticity of adventitious rooting in Arabidopsis is controlled by complex regulation of AUXIN RESPONSE FACTOR transcripts and microRNA abundance. Plant Cell.

[20] Gutierrez L., Mongelard G., Flokova K., Pacurar D.I., Novak O., Staswick P., Kowalczyk M., Pacurar M., Demailly H., Geiss G. (2012). Auxin controls Arabidopsis adventitious root initiation by regulating jasmonic acid homeostasis. Plant Cell.

[21] Mashiguchi K., Tanaka K., Sakai T., Sugawara S., Kawaide H., Natsume M., Hanada A., Yaeno T., Shirasu K., Yao H. (2011). The main auxin biosynthesis pathway in Arabidopsis. Proc. Natl. Acad. Sci. USA.

[22] Li Y.H., Zou M.H., Feng B.H., Huang X., Zhang Z., Sun G.M. (2012). Molecular cloning and characterization of the genes encoding an auxin efflux carrier and the auxin influx carriers associated with the adventitious root formation in mango (*Mangifera indica* L.) cotyledon segments. Plant Physiol. Biochem..

[23] Peret B., Swarup K., Ferguson A., Seth M., Yang Y., Dhondt S., James N., Casimiro I., Perry P., Syed A. (2012). AUX/LAX genes encode a family of auxin influx transporters that perform distinct functions during Arabidopsis development. Plant Cell.

[24] Sukumar P., Maloney G.S., Muday G.K. (2013). Localized induction of the ATP-binding cassette B19 auxin transporter enhances adventitious root formation in Arabidopsis. Plant Physiol..

[25] Pacurar D.I., Perrone I., Bellini C. (2014). Auxin is a central player in the hormone cross-talks that control adventitious rooting. Physiol. Plant.

[26] Bolger A.M., Lohse M., Usadel B. (2014). Trimmomatic: A flexible trimmer for Illumina sequence data. Bioinformatics.

[27] Li S.W., Xue L.G., Xu S.J., Feng H.Y., An L.Z. (2009). Hydrogen peroxide acts as a signal molecule in the adventitious root formation of mung bean seedlings. Environ. Exp. Bot..

[28] Koyuncu F., Balta F. (2004). Adventitious root formation in leaf-bud cuttings of tea (*Camellia sinensis* L.). Pak. J. Bot..

[29] Montain C.R., Haissig B.E., Curtis J.D. (1983). Differentiation of adventitious root primordia in callus of Pinusbanks. Can. J. For. Res..

[30] Goldfarb B., Hackett W.P., Furnier G.R., Mohn C.A., Plietzsch A. (2010). Adventitious root initiation in hypocotyl and epicotyl cuttings of eastern white pine (*Pinus strobus*) seedlings. Physiol. Plant..

[31] Thimann K.V., Went F.W. (1934). On the chemical nature of the root forming hormone. Proc. Kon. Acad. Wetensch. Amst..

[32] Ludwig-Müller J. (2000). Indole-3-butyric acid in plant growth and development. Plant Growth Regulat..

[33] Li Y.H., Zhang H.N., Wu Q.S., Muday G.K. (2017). Transcriptional sequencing and analysis of major genes involved in the adventitious root formation of mango cotyledon segments. Planta.

[34] Druege U., Franken P., Lischewski S., Ahkami A.H., Zerche S., Hause B., Hajirezaei M.R. (2014). Transcriptomic analysis reveals ethylene as stimulator and auxin as regulator of adventitious root formation in petunia cuttings. Front. Plant Sci..

[35] Peat T.S., Bottcher C., Newman J., Lucent D., Cowieson N., Davies C. (2012). Crystal structure of an indole-3-acetic acid amido synthetase from grapevine involved in auxin homeostasis. Plant Cell.

[36] Steffens B., Rasmussen A. (2016). The Physiology of Adventitious Roots. Plant Physiol..

[37] Van Der Krieken W.M., Breteler H., Visser M.H., Mavridou D. (1993). The role of the conversion of IBA into IAA on root regeneration in apple: Introduction of a test system. Plant Cell Rep..

[38] Díaz-Sala C. (2014). Direct reprogramming of adult somatic cells toward adventitious root formation in forest tree species: The effect of the juvenile-adult transition. Front. Plant Sci..

[39] BlažKová A., Sotta B., Tranvan H., Maldiney R., Bonnet M., Einhorn J., Kerhoas L., Miginiac E. (2010). Auxin metabolism and rooting in young and mature clones of Sequoia sempervirens. Physiol. Plant..

[40] Tonon G., Kevers C., Gaspar T. (2001). Changes in polyamines, auxins and peroxidase activity during in vitro rooting of Fraxinus angustifolia shoots: An auxin-independent rooting model. Tree Physiol..

[41] Yang G., Chen S., Wang S., Liu G., Li H., Huang H., Jiang J. (2015). BpGH3.5, an early auxin-response gene, regulates root elongation in *Betula platyphylla* × *Betula pendula*. Plant Cell Tissue Organ Cult..

[42] Cano A., Sánchez-García A.B., Albacete A., González-Bayón R., Justamante M.S., Ibáñez S., Acosta M., Pérez-Pérez J.M. (2018). Enhanced Conjugation of Auxin by GH3 Enzymes Leads to Poor Adventitious Rooting in Carnation Stem Cuttings. Front. Plant Sci..

[43] Jain M., Kaur N., Tyagi A.K., Khurana J.P. (2006). The auxin-responsive GH3 gene family in rice (*Oryza sativa*). Funct. Integr. Genom..

[44] Bollmark M., Kubát B., Eliasson L. (1988). Variation in Endogenous Cytokinin Content during Adventitious Root Formation in Pea Cuttings. J. Plant Physiol..

[45] Gonzalez-Rizzo S., Crespi M., Frugier F. (2006). The Medicago truncatula CRE1 cytokinin receptor regulates lateral root development and early symbiotic interaction with Sinorhizobium meliloti. Plant Cell.

[46] Laplaze L., Benkova E., Casimiro I., Maes L., Vanneste S., Swarup R., Weijers D., Calvo V., Parizot B., Herrera-Rodriguez M.B. (2007). Cytokinins act directly on lateral root founder cells to inhibit root initiation. Plant Cell.

[47] Rani D.B., Taketa S., Ichii M. (2005). Cytokinin inhibits lateral root initiation but stimulates lateral root elongation in rice (*Oryza sativa*). J. Plant Physiol..

[48] Werner T., Motyka V., Laucou V., Smets R., Van Onckelen H., Schmulling T. (2003). Cytokinin-deficient transgenic Arabidopsis plants show multiple developmental alterations indicating opposite functions of cytokinins in the regulation of shoot and root meristem activity. Plant Cell.

[49] Li X., Mo X., Shou H., Wu P. (2006). Cytokinin-mediated cell cycling arrest of pericycle founder cells in lateral root initiation of Arabidopsis. Plant Cell Physiol..

[50] Lo S.F., Yang S.Y., Chen K.T., Hsing Y.I., Zeevaart J.A., Chen L.J., Yu S.M. (2008). A novel class of gibberellin 2-oxidases control semidwarfism, tillering, and root development in rice. Plant Cell.

[51] Lombardi-Crestana S., Da S.A.M., e Silva G.F., Pino L.E., Appezzato-da-Gloria B., Figueira A., Nogueira F.T., Peres L.E. (2012). The tomato (*Solanum lycopersicum* cv. Micro-Tom) natural genetic variation Rg1 and the DELLA mutant procera control the competence necessary to form adventitious roots and shoots. J. Exp. Bot..

[52] Mauriat M., Petterle A., Bellini C., Moritz T. (2014). Gibberellins inhibit adventitious rooting in hybrid aspen and Arabidopsis by affecting auxin transport. Plant J..

[53] Steffens B., Sauter M. (2005). Epidermal cell death in rice is regulated by ethylene, gibberellin, and abscisic acid. Plant Physiol..

[54] Agullo-Anton M.A., Ferrandez-Ayela A., Fernandez-Garcia N., Nicolas C., Albacete A., Perez-Alfocea F., Sanchez-Bravo J., Perez-Perez J.M., Acosta M. (2014). Early steps of adventitious rooting: Morphology, hormonal profiling and carbohydrate turnover in carnation stem cuttings. Physiol. Plant.

[55] Vidoz M.L., Loreti E., Mensuali A., Alpi A., Perata P. (2010). Hormonal interplay during adventitious root formation in flooded tomato plants. Plant J..

[56] Geiss G., Gutierrez L., Bellini C. (2009). Adventitious Root Formation: New Insights and Perspectives.

[57] Kurepin L., Haslam T., Lopez-Villalobos A., Oinam G., Yeung E. (2011). Adventitious root formation in ornamental plants: II. The role of plant growth regulators. Propag. Ornam. Plants.

[58] Müssig C., Shin G.H., Altmann T. (2003). Brassinosteroids Promote Root Growth in Arabidopsis. Plant Physiol..

[59] Bao F., Shen J., Brady S.R., Muday G.K., Asami T., Yang Z. (2004). Brassinosteroids interact with auxin to promote lateral root development in Arabidopsis. Plant Physiol..

[60] Mouchel C.F., Briggs G.C., Hardtke C.S. (2004). Natural genetic variation in Arabidopsis identifies BREVIS RADIX, a novel regulator of cell proliferation and elongation in the root. Genes Dev..

[61] Nemhauser J.L., Mockler T.C., Chory J. (2004). Interdependency of brassinosteroid and auxin signaling in Arabidopsis. PLoS Biol..

[62] Yong H.C., Chang H.S., Gupta R., Wang X., Zhu T., Luan S. (2002). Transcriptional Profiling Reveals Novel Interactions between Wounding, Pathogen, Abiotic Stress, and Hormonal Responses in Arabidopsis. Plant Physiol..

[63] Yang L., Conway S.R., Poethig R.S. (2011). Vegetative phase change is mediated by a leaf-derived signal that represses the transcription of miR156. Development.

[64] Emons A.M.C. (1994). Somatic embryogenesis: Cell biological aspects. Plant Biol..

[65] Klerk G.J.D., Keppel M., Brugge J.T., Meekes H. (1995). Timing of the phases in adventitious root formation in apple microcuttings. J. Exp. Bot..

[66] Pawlicki N., Welander M. (1995). Influence of carbohydrate source, auxin concentration and time of exposure on adventitious rooting of the apple rootstock Jork 9. Plant Sci..

[67] Calamar A., Klerk G.J.D. (2002). Effect of sucrose on adventitious root regeneration in apple. Plant Cell Tissue Organ Cult..

[68] Koch K. (2004). Sucrose metabolism: Regulatory mechanisms and pivotal roles in sugar sensing and plant development. Curr. Opin. Plant Biol..

[69] Rolland F., Baena-Gonzalez E., Sheen J. (2006). Sugar sensing and signaling in plants: Conserved and novel mechanisms. Annu. Rev. Plant Biol..

[70] Appeldoorn N.J., Sergeeva L., Vreugdenhil D., van Der Plas L.H., Visser R.G. (2002). In situ analysis of enzymes involved in sucrose to hexose-phosphate conversion during stolon-to-tuber transition of potato. Physiol. Plant.

[71] Kromer K., Gamian A. (2000). Analysis of soluble carbohydrates, proteins and lipids in shoots of M7 apple rootstock cultured in vitro during regeneration of adventitious roots. J. Plant Physiol..

[72] Smeekens S., Ma J., Hanson J., Rolland F. (2010). Sugar signals and molecular networks controlling plant growth. Curr. Opin. Plant Biol..

[73] Moreno F., Ahuatzi D., Riera A., Palomino C.A., Herrero P. (2005). Glucose sensing through the Hxk2-dependent signalling pathway. Biochem. Soc. Trans..

[74] Zhong C., Kai G., Su X., Rao P., An X. (2015). Genome-Wide Identification of the Invertase Gene Family in Populus. PLoS ONE.

[75] Dello I.R., Linhares F.S., Scacchi E., Casamitjana-Martinez E., Heidstra R., Costantino P., Sabatini S. (2007). Cytokinins determine Arabidopsis root-meristem size by controlling cell differentiation. Curr. Biol..

[76] De Smet I., Tetsumura T., De Rybel B., Frei D.F.N., Laplaze L., Casimiro I., Swarup R., Naudts M., Vanneste S., Audenaert D. (2007). Auxin-dependent regulation of lateral root positioning in the basal meristem of Arabidopsis. Development.

[77] Himanen K., Boucheron E., Vanneste S., de Almeida E.J., Inze D., Beeckman T. (2002). Auxin-mediated cell cycle activation during early lateral root initiation. Plant Cell.

[78] Wolters H., Jurgens G. (2009). Survival of the flexible: Hormonal growth control and adaptation in plant development. Nat. Rev. Genet..

[79] Link M., Rausch T., Greiner S. (2004). In Arabidopsis thaliana, the invertase inhibitors AtC/VIF1 and 2 exhibit distinct target enzyme specificities and expression profiles. FEBS Lett..

[80] Smith D.L., Fedoroff N.V. (1995). LRP1, a gene expressed in lateral and adventitious root primordia of arabidopsis. Plant Cell.

[81] Li B., Wang J., Ren X., Bao L., Zhang L., Zhang L., Han M., Zhan D. (2015). Root growth, yield and fruit quality of ‘Red Fuji’ apple trees in relation to planting depth of dwarfing interstock on the Loess Plateau. Eur. J. Horticult. Sci..

[82] Cvrckova F., Bezvoda R., Zarsky V. (2010). Computational identification of root hair-specific genes in Arabidopsis. Plant Signal. Behav..

[83] Xu Q., Chai F., An X., Han S. (2012). Production Method for Paraffin Section of Invasive Species of *Bemisia tabaci*. Plant Dis. Pests.

[84] Yang J.P. (2006). Improvement of traditional paraffin section preparation methods. J. Biol..

[85] Weiler E.W., Jourdan P.S., Conrad W. (1981). Levels of indole-3-acetic acid in intact and decapitated coleoptiles as determined by a specific and highly sensitive solid-phase enzyme immunoassay. Planta.

[86] Fan S., Zhang D., Lei C., Chen H.F., Xing L.B., Ma J.J., Zhao C.P., Han M.Y. (2016). Proteome analyses using itraq labeling reveals critical mechanisms in alternate bearing malus prunifolia. J. Proteome Res..

[87] Gambino G., Perrone I., Gribaudo I. (2008). A Rapid and effective method for RNA extraction from different tissues of grapevine and other woody plants. Phytochem. Anal..

[88] Qian L., Liu Y., Qi Y., Jiao S., Tian F., Jiang L., Wang Y. (2014). Transcriptome sequencing and metabolite analysis reveals the role of delphinidin metabolism in flower colour in grape hyacinth. J. Exp. Bot..

[89] Kim D., Langmead B., Salzberg S.L. (2015). HISAT: A fast spliced aligner with low memory requirements. Nat. Methods.

[90] Kong L., Zhang Y., Ye Z.Q., Liu X.Q., Zhao S.Q., Wei L., Gao G. (2007). CPC: Assess the protein-coding potential of transcripts using sequence features and support vector machine. Nucleic Acids Res..

[91] Frazee A.C., Pertea G., Jaffe A.E., Langmead B., Salzberg S.L., Leek J.T. (2014). Flexible isoform-level differential expression analysis with Ballgown. Biorxiv.

[92] Alexa A., Rahnenfuhrer J. (2006). topGO: Enrichment Analysis for Gene Ontology.

[93] Xie C., Mao X., Huang J., Ding Y., Wu J., Dong S., Kong L., Gao G., Li C.Y., Wei L. (2011). KOBAS 2.0: A web server for annotation and identification of enriched pathways and diseases. Nucleic Acids Res..

[94] Livak K.J., Schmittgen T.D. (2001). Analysis of relative gene expression data using real-time quantitative PCR and the 2^−ΔΔ*C*t^ Method. Methods.

[95] Shalom L., Samuels S., Zur N., Shlizerman L., Doron-Faigenboim A., Blumwald E., Sadka A. (2014). Fruit load induces changes in global gene expression and in abscisic acid (ABA) and indole acetic acid (IAA) homeostasis in citrus buds. J. Exp. Bot..

