# Supplementary material for: Transcriptome Analysis Reveals Multiple Hormones, Wounding and Sugar Signaling Pathways Mediate Adventitious Root Formation in Apple Rootstock
Source: Int J Mol Sci. 2018 Jul 27;19(8):2201. doi: 10.3390/ijms19082201 (PMC6121287; doi:10.3390/ijms19082201)
Supplement: Supplementary file 1 [file ijms-19-02201-s001.zip › ijms-289143-SI.pdf]

Supplementary

Transcriptome Analysis Reveals Multiple Hormones, Wounding and Sugar Signaling Pathways Mediate Adventitious Root Formation in Apple Rootstock

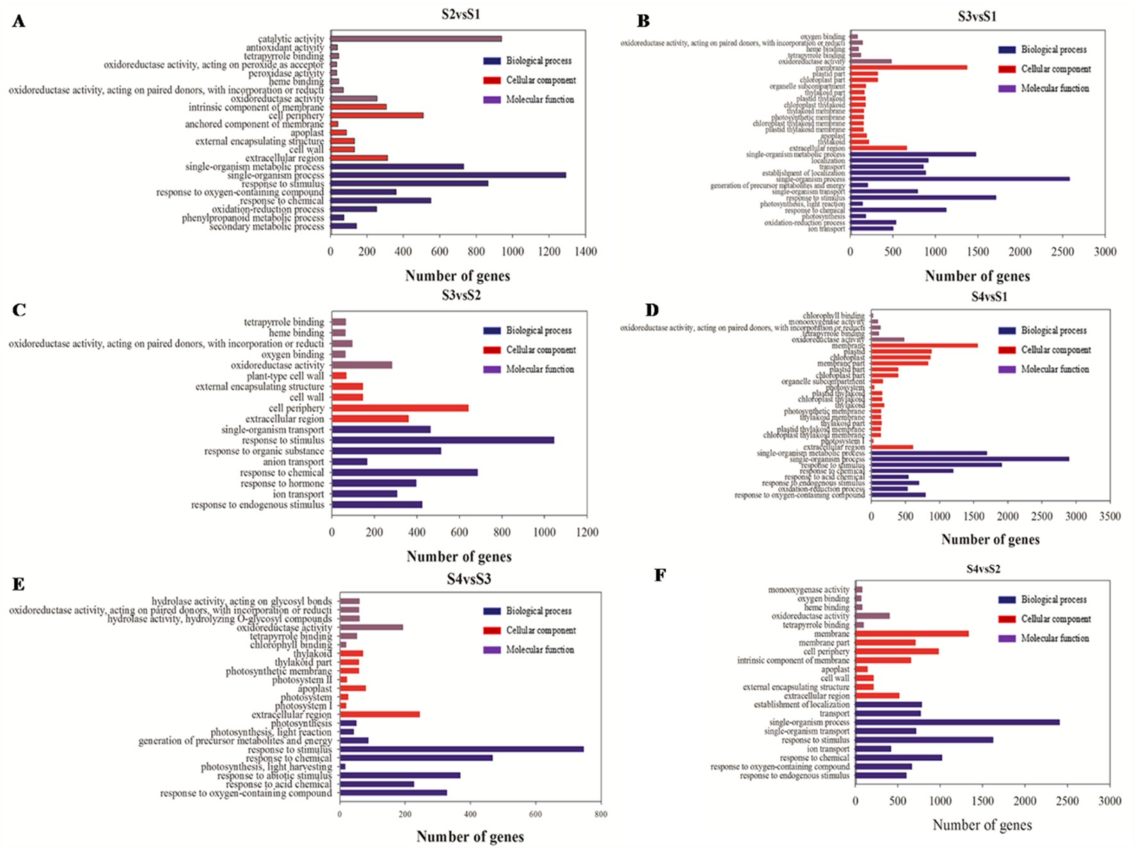

Figure S1. GO classification of the DEGs between the different stages (S1, S2, S3, and S4) of adventitious root (AR) formation. A: GO classification of the DEGs in S1 vs. S2. B: GO classification of the DEGs in S3 vs. S1. C: GO classification of the DEGs in S3 vs. S2. D: GO classification of the DEGs in S4 vs. S1. E: GO classification of the DEGs in S4 vs. S3. F: GO classification of the DEGs in S4 vs. S2. The blue bars indicate biological process; the red bars indicate cellular component; the purple bars indicate molecular function.

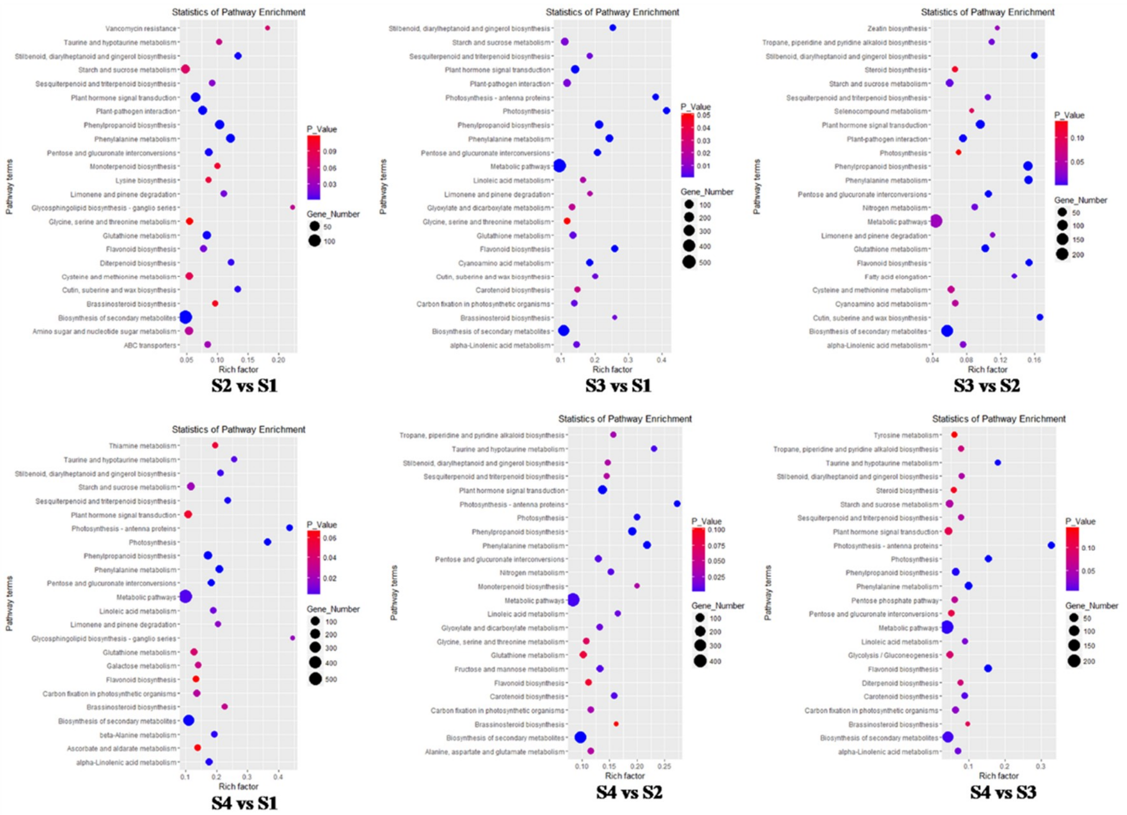

Figure S2. Differentially Expressed Genes (DEGs) KEGG Pathway Enrichment Analysis. The ordinate indicates the enriched pathway name, the abscissa represents ‘Rich factor’ (Sample number / Background number), ‘dot size’ indicates the relative number of DEGs in this pathway, while the color of the dots corresponds to a range of different P-values after correction.

Table S1. Sequence of primers used in the RT-qPCR analyses to validate gene expression.

Genes	Gene ID	Forward (5' to 3')	Reverse (5' to 3')
IPT1-2	MDP0000189484	GAGCATACGAAGAGGCGGTGAG	GCGTCCGTTGCGTCTAGTCT
IPT5	MDP0000220668	GCGGATAGTGCGTCAGTTCCTT	AGCGGTCACTGTCGGCATAGA
CRF4	MDP0000308379	CGAGGTGGAGACTAACGGAACGA	GAAGTGGAGGACGGAGGTTGGA
CKX7	MDP0000238100	TGTCTCGAACTCGCTCGTCACT	CCACCTGGAACTTCAACTGCTCAA
CKX	MDP0000223673	TGCCACACCAGCCACTCCAA	TGCTGCGTGATTGTTGTTGTTGAA
GATA1	MDP0000190038	AGATGAGGATGATGCGGAAGATGT	CTCCTAATTGCGATGATGGATGCT
GATA5	MDP0000172464	TCACCATCATCATCGCCTTGTTCT	GCAATGACTACACCTCCGCTGAA
ARF11	MDP0000268306	ACCAGCAGCAGCAGCAATTAGTT	TGGCATAGCGATGTGACGACTTG
ARF9	MDP0000634433	GTCCACAGAACAAATGGGCAGTTG	CATCTTCTGTACCTCGTCGCTTGA
ARF1	MDP0000564492	ATTGCTGCTCATACTCCTCCTCCT	GTGCTGCTGCTGCTGACCTT
IAA29	MDP0000543718	CAGCAGCAGCAGCGAATAATTCC	CAAGGTGTCTCTGAGCGACTGAA
IAA19	MDP0000296324	GGCATCGGCGAGGTGTTGAA	CCACTAGCATCCAGTCTCCATCTT
PIN1	MDP0000138035	TGATGCCTCCAACAAGTGTGATGA	GAACGTGCCACCTGAATGAGACTA
AXR3/IAA17	MDP0000253285	CGGACTGAACTACGACGAGACCAA	TCACAACATTCCACCCTTGCTCCT
IAA3/SHY2	MDP0000303142	AGCATGGATGGAGCACCTTACCT	ATCCTTTGTAGCCCTCCCTCTCTG
PLT2	MDP0000871080	ATCCGCCACTACTACTGCTCCTAA	AGTTGGTGCTATCCGATGAGTTCA
BR6OX2	MDP0000286141	GACTCTGCCTGCCGATGCTT	GGTTCTTGATGTGTTGCTCCAGTG
JAZ12	MDP0000901967	TACCAAGCCACCAACTCCTGTCA	CAGCACGTCATGCAAGCTCTCA
JAZ26	MDP0000565690	AACCATGAACTTGCTGACGACCAT	TCCAGGCTGCTGATCTCCCAAA
GA2OX4	MDP0000161181	ACTCAGTTGTGAAGCCAAGGATGT	CGGCGACACCATCTCTGGAAGA
GA2OX2	MDP0000145827	ACATCCTCCTCAGCACCAATCTTG	ATCAGCCACCATTTCCAGCACTT
GH3.1	MDP0000121609	AGACGACCAGGAACACGGACTC	TTGAGGTATTCGGTCTCGGCGTTA
PAT1	MDP0000152670	TGGCACAATGGATGAAGGATGAGT	AGTTCGGACCTCGCTGGTTCTT
SAMS3	MDP0000302980	TCTGTTCACCGTCACTATCTCACC	GCACCACTCCTGTCCACCTT
ACO1	MDP0000200896	TCCACCATCCAAGAACAACACCAT	GCATCGCCAGCAGGATTGTAGAA
EFE1	MDP0000251295	TGGACACAGTGGAGAGGCTGAC	ACTTCTGTCTGAACGGCATCAAGG
EFE2	MDP0000200737	CGCTGGTGGTATCATCCTGCTT	CATTCTGGTTCCGTCCGACTGAG
ERF1	MDP0000235313	AAGGATGTTCGCCGGTGATGG	CGCTGGTACTTGTATTCTCGGTTG
LRP1	MDP0000171430	TCTCCTCTCCTCCTCCGACCAT	AATGACACCGACGCCGACAC
SHI	MDP0000147890	ACCAACACCACCAACAGCAAGAT	TGCGATGTTCACAGCCGTCTG
SCR1	MDP0000203826	TCCTTATGCGGTGACTCTGTCCAA	ACTCTTCTTCACTCGTCGGTTCCT
SCL5	MDP0000284679	ATCCTCGGGAACCAGTGCCTAC	GTTGACAGGAGAGCCACCAGACT
RHS19	MDP0000488361	CCACCACCACCTTCATCATCATCA	CAGCCTCAGTGAGTCCACAGTTC
MDH	MDP0000277049	GAGATGCTGGCGTTGTTGAATGC	CCTTCTGGATGCTTGCTGCTAACT
FLS1	MDP0000294667	CGAGCAAGCCAACTCTGAACGA	AAGACGCCACTGATGGATCTACTG
CYCD1;1	MDP0000809276	GCCTGATGATACCACCACCACCTC	GCGGAACCTGGAGATGTAGGATGG
CYCD3;1	MDP0000286130	GCGGTGGATTGGATGCTGAGAG	GAAGTTGGAGGCTGGACAAGAACC
CYCP4;1	MDP0000139550	CCGTCTTGCTTAATTGTTGCGTAC	GCAGCCACCATGACACTCGTA
Wd-Id-4	MDP0000836784	GTGGCAGCAAGTATTGGAGCAGTT	GCAGAAGACGAAGAGTCGGAGAGT
Wd-Id-8	MDP0000228919	TCTCTGGCGGCGAAGCATCA	TGGAGGGACTCGTCAGTTTGGTT
WRKY	MDP0000602139	GAAGCAAGTGGAGCGGAACAGAT	CGAGCGAGTTGCGGTGAGTT
SUS4-3	MDP0000250070	AGGACAATGAGGAGAAGGCTGAGA	ACCATTCCTGACACGGTTCATCTG
SPS4F	MDP0000288684	ATGCCTGTATGGTCCGATGATGTT	CGAGAGCACGAGCAAGTTCCA
WINV4	MDP0000275150	TGCGTTCCGAGACCCGACAA	GGAGCAGAGTGGAGAGGATGATGG

HXK2	MDP0000181206	GCGGTTGAGCAAGATGTTGTAGG	CATAAGCAGCGTTCGTTCCAGTG
PMT5	MDP0000688348	AGTCACCTCGTTGGCTCGTCAT	ACCGTGGCTGCGTTTAGATACCT
C/VIF1	MDP0000305934	TACTCATTGCACCGCCAACAACC	AGGAGAGCGTCGGATGCTATCAG
	ACTIN	TGACCGAATGAGCAAGGAAATTACT	TACTCAGCTTTGGCAATCCACATC

Table S2. Summary of the sequencing statistics obtained for each of the samples subjected to RNA-seq using an Illumina platform.

	S1_1	S1_2	S1_3
total reads	29086044	23838464	18063618
aligned concordantly 0 times	11493957 (39.52%)	10438558 (43.79%)	8048133 (44.55%)
aligned concordantly exactly 1 time	13172963 (45.29%)	9805199 (41.13%)	7341406 (40.64%)
aligned concordantly >1 times	4419124 (15.19%)	3594707 (15.08%)	2674079 (14.80%)
overall alignment rate	65.69%	62.37%	61.60%
	**S2_1**	**S2_2**	**S2_3**
total reads	32705777	22902663	24102594
aligned concordantly 0 times	12139092 (37.12%)	10458893 (45.67%)	10559313 (43.81%)
aligned concordantly exactly 1 time	15366115 (46.98%)	9056595 (39.54%)	9866985 (40.94%)
aligned concordantly >1 times	5200570 (15.90%)	3387175 (14.79%)	3676296 (15.25%)
overall alignment rate	68.44%	60.72%	62.29%
	**S3_1**	**S3_2**	**S3_3**
total reads	35263599	26986857	29754876
aligned concordantly 0 times	13005583 (36.88%)	11360125 (42.10%)	12612850 (42.39%)
aligned concordantly exactly 1 time	16490294 (46.76%)	11412094 (42.29%)	12526298 (42.10%)
aligned concordantly >1 times	5767722 (16.36%)	4214638 (15.62%)	4615728 (15.51%)
overall alignment rate	68.32%	63.72%	63.20%
	**S4_1**	**S4_2**	**S4_3**
total reads	27156578	26008927	28352841
aligned concordantly 0 times	10227320 (37.66%)	11307666 (43.48%)	12053023 (42.51%)
aligned concordantly exactly 1 time	12465020 (45.90%)	10638407 (40.90%)	11758334 (41.47%)
aligned concordantly >1 times	4464238 (16.44%)	4062854 (15.62%)	4541484 (16.02%)
overall alignment rate	67.81%	62.75%	63.38%

Table S3. Summary of the sequencing statistics for the clean reads obtained in each sample.

Filename	Total Sequences	Sequence length	%GC	Total Percentage	Q20(%)	Q30(%)
S1_1	29086044	114-126	47	50.71156097	99.340756	93.58549069
S1_1	29086044	114-126	47	50.71424921	98.92312616	91.36095304
S2_1	32705777	114-126	47	48.79971331	99.34858909	93.53149139
S2_1	32705777	114-126	47	48.21483689	98.82456852	90.74312774
S3_1	35263599	114-126	48	44.98571313	99.29397167	93.12435183
S3_1	35263599	114-126	48	46.8170726	98.84582399	90.71421496
S4_1	27156578	114-126	48	49.47068727	99.2784842	92.95830277
S4_1	27156578	114-126	48	49.22643361	98.81154393	90.44684496
S1_2	23838464	114-126	47	55.94454853	98.89387588	90.32083191
S1_2	23838464	114-126	47	57.82722839	97.90658492	85.84029995
S2_2	22902663	114-126	48	52.48166846	98.77958297	89.90841371
S2_2	22902663	114-126	48	54.35704044	97.87359662	86.07480711
S3_2	26986857	114-126	47	55.75719092	98.88379369	90.4081346
S3_2	26986857	114-126	47	57.69937997	97.77695861	85.45134396
S4_2	26008927	114-126	47	52.70288728	98.70210332	89.72893422
S4_2	26008927	114-126	47	53.80187346	97.82119808	85.95887866
S1_3	18063618	114-126	47	58.9415796	98.90929934	90.45063951
S1_3	18063618	114-126	47	60.57150036	97.81503351	85.50723338
S2_3	24102594	114-126	47	54.25026432	98.84219516	90.28373461
S2_3	24102594	114-126	47	56.43160791	97.81534718	85.7757136
S3_3	29754876	114-126	47	52.38728383	98.80175269	90.00838048
S3_3	29754876	114-126	47	53.65553914	98.03292408	86.67356906
S4_3	28352841	114-126	48	51.46086439	98.92881281	90.42226492
S4_3	28352841	114-126	48	53.09013645	97.76057715	85.21974923

Table S4. Selection of auxin-related genes from among the DEGs identified by RNA-seq analysis of stem cuttings of 'T337' apple rootstocks during AR formation. The colors in every other column indicate different gene expression levels.

Apple genes Identification	Arabidopsis Homolog	Annotation	S1 FPKM	S1 Log2 (FPKM)	S2 FPKM	S2 Log2 (FPKM)	S3 FPKM	S3 Log2 (FPKM)	S4 FPKM	S4 Log2 (FPKM)
**IAA related**										
MDP0000634433	AT4G23980.1	auxin response factor 9 (ARF9)	2.77	1.47	2.15	1.11	9.84	3.30	9.60	3.26
MDP0000564492	AT1G59750.1	auxin response factor 1 (ARF1)	0.13	-2.98	1.04	0.06	1.32	0.40	1.47	0.55
MDP0000268306	AT1G19220.1	auxin response factor 19 (ARF11)	34.53	5.11	30.20	4.92	19.16	4.26	14.68	3.88
MDP0000232116	AT1G77850.1	auxin response factor 17 (ARF17)	0.75	-0.41	1.77	0.82	3.73	1.90	3.04	1.61
MDP0000497581	AT1G70940.1	Auxin efflux carrier family protein (PIN3)	0.00	-14.01	0.00	-13.63	0.27	-1.90	0.01	-7.63
MDP0000138035	AT1G73590.1	Auxin efflux carrier family protein (PIN1)	32.61	5.03	22.86	4.51	20.35	4.35	10.07	3.33
MDP0000259421	AT1G15520.1	pleiotropic drug resistance 12 (PDR12)	5.09	2.35	4.58	2.19	2.85	1.51	2.05	1.03
MDP0000257483	AT2G26910.1	pleiotropic drug resistance 4 (PDR4)	1.38	0.47	0.42	-1.25	0.15	-2.76	0.00	-0.49
MDP0000253285	AT1G04250.1	AUX/IAA transcriptional regulator family protein (IAA17)	11.86	3.57	20.06	4.33	23.32	4.54	33.16	5.05
MDP0000324398	AT5G43700.1	AUX/IAA transcriptional regulator family protein (IAA4)	1.81	0.86	9.41	3.23	5.04	2.33	1.18	0.24
MDP0000876321	AT1G19220.1	auxin response factor 19 (ARF19)	4.40	2.14	5.34	2.42	4.64	2.21	4.30	2.10
MDP0000303142	AT1G04240.1	AUX/IAA transcriptional regulator family protein(IAA3/SHY2)	13.48	3.75	16.93	4.08	23.04	4.53	15.22	3.93
MDP0000543718	AT4G32280.1	indole-3-acetic acid inducible 29 (IAA29)	5.88	2.56	6.20	2.63	0.47	-1.10	0.17	-2.52
MDP0000295589	AT1G04240.1	AUX/IAA transcriptional regulator family protein(IAA3/SHY2)	1.81	0.85	2.11	1.08	1.86	0.90	0.28	-1.83
MDP0000226842	AT1G28130.1	Auxin-responsive GH3 family protein (GH3.17)	0.04	-4.51	0.17	-2.60	2.05	1.03	0.57	-0.82
MDP0000121609	AT2G14960.1	Auxin-responsive GH3 family protein (GH3.1)	0.09	-3.42	28.41	4.83	17.78	4.15	10.80	3.43
MDP0000132162	AT5G54510.1	Auxin-responsive GH3 family protein (GH3.6)	3.58	1.84	7.74	2.95	14.01	3.81	13.55	3.76
MDP0000204381	AT2G47750.1	putative indole-3-acetic acid-amido synthetase GH3.9(GH3.9)	0.64	-0.65	0.11	-3.20	0.33	-1.61	0.35	-1.53
MDP0000226842	AT1G28130.1	Auxin-responsive GH3 family protein (GH3.17)	0.04	-4.51	0.17	-2.60	2.05	1.03	0.57	-0.82
MDP0000568498	AT2G47750.1	putative indole-3-acetic acid-amido synthetase GH3.9(GH3.9)	5.38	2.43	0.39	-1.37	1.75	0.81	1.16	0.22
MDP0000612660	AT2G14960.1	Auxin-responsive GH3 family protein (GH3.1)	1.29	0.37	45.42	5.51	29.51	4.88	16.13	4.01
MDP0000811081	AT4G03400.1	Auxin-responsive GH3 family protein (GH3.10)	1.57	0.65	0.34	-1.56	0.09	-3.44	0.10	-3.34
MDP0000204381	AT2G47750.1	putative indole-3-acetic acid-amido synthetase GH3.9(GH3.9)	0.64	-0.65	0.11	-3.20	0.33	-1.61	0.35	-1.53
MDP0000333431	AT3G16500.1	phytochrome-associated protein 1 (PAP1)	2.24	1.17	5.32	2.41	7.07	2.82	8.09	3.02
MDP0000296324	AT3G15540.1	indole-3-acetic acid inducible 19 (IAA19,MSG2)	3.94	1.98	16.73	4.06	4.94	2.31	3.73	1.90
MDP0000237499	AT3G23050.1	indole-3-acetic acid 7 (IAA3)	0.24	-2.09	15.39	3.94	14.33	3.84	15.32	3.94
MDP0000195460	AT4G32280.1	indole-3-acetic acid inducible 29 (IAA29)	4.58	2.19	14.43	3.85	7.74	2.95	1.19	0.26
MDP0000367919	AT2G46690.1	SAUR-like auxin-responsive protein family	39.78	5.31	62.91	5.98	86.44	6.43	83.79	6.39
MDP0000315517	AT2G37030.1	SAUR-like auxin-responsive protein family	0.10	-3.32	0.15	-2.70	0.76	-0.39	14.03	3.81
MDP0000288641	AT1G75590.1	SAUR-like auxin-responsive protein family	0.22	-2.20	0.51	-0.96	1.61	0.69	2.63	1.39
MDP0000286931	AT5G50760.1	SAUR-like auxin-responsive protein family	8.78	3.13	22.10	4.47	185.30	7.53	232.49	7.86
MDP0000183569	AT3G12830.1	SAUR-like auxin-responsive protein family	0.37	-1.42	0.10	-3.27	0.28	-1.85	3.58	1.84

MDP0000153382	AT2G24400.1	SAUR-like auxin-responsive protein family	3.54	1.82	30.92	4.95	38.40	5.26	47.77	5.58
MDP0000141719	AT1G75580.1	SAUR-like auxin-responsive protein family	4.53	2.18	21.55	4.43	16.99	4.09	17.81	4.15
MDP0000596615	AT1G72430.1	SAUR-like auxin-responsive protein family	61.59	5.94	30.93	4.95	37.77	5.24	152.98	7.26
MDP0000912039	AT2G21220.1	SAUR-like auxin-responsive protein family	9.36	3.23	13.02	3.70	5.79	2.53	2.40	1.26
MDP0000874044	AT2G24400.1	SAUR-like auxin-responsive protein family	0.06	-4.07	1.40	0.49	21.67	4.44	8.30	3.05
MDP0000848515	AT3G12830.1	SAUR-like auxin-responsive protein family	26.44	4.72	18.02	4.17	26.20	4.71	51.93	5.70
MDP0000828073	AT2G36210.1	SAUR-like auxin-responsive protein family	3.49	1.80	0.56	-0.84	0.46	-1.11	0.10	-3.32
MDP0000654738	AT4G34800.1	SAUR-like auxin-responsive protein family	42.24	5.40	34.41	5.10	17.17	4.10	13.46	3.75
MDP0000810244	AT5G50760.1	SAUR-like auxin-responsive protein family	26.26	4.71	20.48	4.36	53.93	5.75	74.52	6.22
MDP0000786165	AT2G46690.1	SAUR-like auxin-responsive protein family	16.52	4.05	14.44	3.85	57.61	5.85	44.22	5.47
MDP0000313042	AT5G55090.1	mitogen-activated protein kinase15 (MAPKKK15)	4.66	2.22	4.83	2.27	2.97	1.57	0.54	-0.89
MDP0000217310	AT2G30040.1	mitogen-activated protein kinase 14 (MAPKKK14)	2.91	1.54	1.58	0.66	3.95	1.98	4.01	2.00
MDP0000187103	AT5G67080.1	mitogen-activated protein kinase 19 (MAPKKK19)	10.06	3.33	3.47	1.80	10.31	3.37	16.30	4.03
MDP0000193927	AT5G55090.1	mitogen-activated protein kinase15 (MAPKKK15)	1.11	0.15	0.80	-0.33	3.72	1.90	4.74	2.24
MDP0000431417	AT1G07150.1	mitogen-activated protein kinase 13 (MAPKKK13)	1.76	0.82	0.49	-1.02	0.67	-0.59	0.54	-0.90
MDP0000835932	AT5G67080.1	mitogen-activated protein kinase 19 (MAPKKK19)	16.46	4.04	14.79	3.89	15.19	3.92	6.76	2.76
MDP0000203826	AT3G54220.1	GRAS family transcription factor (SCR,SGR1)	0.56	-0.84	3.41	1.77	4.93	2.30	3.81	1.93
MDP0000256486	AT5G66770.1	GRAS family transcription factor (AT5G66770)	9.96	3.32	10.17	3.35	12.48	3.64	8.80	3.14
MDP0000287040	AT2G29060.1	GRAS family transcription factor (AT2G29060)	3.80	1.92	4.71	2.23	2.47	1.30	1.78	0.84
MDP0000152670	AT5G48150.1	GRAS family transcription factor (PAT1)	9.76	3.29	12.95	3.70	5.14	2.36	3.72	1.89
MDP0000308434	AT3G13840.1	GRAS family transcription factor (AT3G13840）	4.24	2.08	8.26	3.05	11.27	3.49	17.68	4.14
MDP0000397638	AT3G54220.1	GRAS family transcription factor (SCR,SGR1）	3.69	1.88	2.51	1.33	7.98	3.00	6.51	2.70
MDP0000190889	AT4G37750.1	Integrase-type DNA-binding superfamily Protein (ANT,CKC.DRG)	3.04	1.61	6.22	2.64	2.26	1.18	1.93	0.95
MDP0000871080	AT1G51190.1	Integrase-type DNA-binding superfamily protein (PLT2)	0.05	-4.19	0.05	-4.38	1.49	0.58	1.93	0.95
MDP0000894037	AT2G28380.1	dsRNA-binding protein 2 (DRB2)	13.77	3.78	18.73	4.23	11.58	3.53	11.82	3.56
MDP0000284679	AT1G50600.1	scarecrow-like 5 (SCL5)	9.79	3.29	7.88	2.98	5.33	2.41	2.80	1.48
MDP0000885425	AT2G21050.1	like AUXIN RESISTANT 2 (LAX2)	7.54	2.91	3.18	1.67	3.11	1.64	2.55	1.35
MDP0000893203	AT1G72770.1	homology to ABI1 (HAB1)	0.72	-0.47	1.76	0.82	2.96	1.57	4.10	2.04
MDP0000195666	AT1G33410.1	SUPPRESSOR OF AUXIN RESISTANCE1 (SAR1)	1.18	0.24	0.35	-1.50	0.59	-0.77	0.58	-0.78

Table S5. Selection of root development- and cell-cycle-related genes from among the DEGs identified by RNA-seq analysis of stem cuttings of 'T337' apple root stocks during AR formation. The colors in every other column indicate different gene expression levels.

Apple genes Identification	Arabidopsis Homolog	Annotation	S1 FPKM	S1 Log2 (FPKM)	S2 FPKM	S2 Log2 (FPKM)	S3 FPKM	S3 Log2 (FPKM)	S4 FPKM	S4 Log2 (FPKM)
**Cell cycle related**										
MDP0000809276	AT1G70210.1	CYCLIN D1;1 (CYCD1;1)	0.91	-0.14	2.72	1.44	7.16	2.84	11.62	3.54
MDP0000304147	AT1G20610.1	Cyclin B2;3 (CYCB2;3)	1.55	0.63	4.47	2.16	5.00	2.32	2.33	1.22
MDP0000286130	AT4G34160.1	CYCLIN D3;1 (CYCD3;1)	26.66	4.74	41.48	5.37	50.14	5.65	45.13	5.50
MDP0000139550	AT2G44740.1	cyclin p4;1 (CYCP4;1)	0.96	-0.05	0.35	-1.51	1.94	0.96	4.62	2.21
MDP0000135761	AT5G45190.1	Cyclin family protein (AT5G45190)(CYCP4;2)	3.56	1.83	4.63	2.21	3.52	1.82	2.59	1.37
MDP0000869524	AT3G05330.1	cyclin family (ATN)	0.85	-0.23	2.64	1.40	2.48	1.31	1.31	0.39
MDP0000275150	AT2G36190.1	cell wall invertase 4 (CWINV4)	7.87	2.98	3.38	1.75	1.62	0.70	1.22	0.29
MDP0000276729	AT1G18040.1	cyclin-dependent kinase D1;3 (CDKD1;3)	4.42	2.14	4.14	2.05	3.32	1.73	2.99	1.58
MDP0000304147	AT1G20610.1	Cyclin B2;3 (CYCB2;3)	1.55	0.63	4.47	2.16	5.00	2.32	2.33	1.22
MDP0000196672	AT1G21270.1	wall-associated kinase 2 (WAK2)	0.32	-1.62	0.03	-5.31	0.26	-1.95	0.00	-15.68
MDP0000186304	AT1G21210.1	wall associated kinase 4 (WAK4)	0.51	-0.98	0.16	-2.68	0.00	-14.31	0.00	-10.96
MDP0000183195	AT1G16120.1	wall associated kinase-like 1 (WAKL1)	7.80	2.96	1.18	0.24	5.14	2.36	3.81	1.93
MDP0000635733	AT4G33270.1	Transducin family protein / WD-40 repeat family protein (CDC20.1)	3.31	1.73	6.82	2.77	7.48	2.90	4.77	2.25
MDP0000194231	AT4G33270.1	Transducin family protein / WD-40 repeat family protein (CDC20.1)	2.14	1.10	7.35	2.88	8.41	3.07	3.49	1.80
MDP0000154241	AT1G19780.1	cyclic nucleotide gated channel 8 (CNGC8)	35.33	5.14	73.52	6.20	28.64	4.84	8.02	3.00
MDP0000119010	AT3G55270.1	mitogen-activated protein kinase phosphatase 1 (MKP1)	5.57	2.48	9.99	3.32	5.63	2.49	4.65	2.22
MDP0000305934	AT1G47960.1	cell wall / vacuolar inhibitor of fructosidase 1 (C/VIF1)	14.77	3.88	33.10	5.05	17.42	4.12	44.54	5.48
**Adventitious root development related**										
MDP0000147890	AT5G66350.1	Lateral root primordium (LRP) protein-related (SHI)	0.20	-2.35	1.81	0.86	4.19	2.07	2.96	1.57
MDP0000154312	AT4G38390.1	root hair specific 17 (RHS17)	0.29	-1.80	0.03	-5.31	0.13	-2.93	0.00	-15.57
MDP0000171430	AT5G12330.4	Lateral root primordium (LRP) protein-related (LRP1)	3.34	1.74	20.55	4.36	30.95	4.95	42.61	5.41
MDP0000203826	AT3G54220.1	GRAS family transcription factor (SCR,SGR1)	0.56	-0.84	3.41	1.77	4.93	2.30	3.81	1.93
MDP0000204381	AT2G47750.1	putative indole-3-acetic acid-amido synthetase GH3.9（GH3.9）	0.64	-0.65	0.11	-3.20	0.33	-1.61	0.35	-1.53
MDP0000226842	AT1G28130.1	Auxin-responsive GH3 family protein (GH3.17)	0.04	-4.51	0.17	-2.60	2.05	1.03	0.57	-0.82
MDP0000313476	AT1G78080.1	related to AP2.4 (RAP2.4)	91.68	6.52	148.33	7.21	317.96	8.31	173.44	7.44
MDP0000397638	AT3G54220.1	GRAS family transcription factor (SCR,SGR1)	3.69	1.88	2.51	1.33	7.98	3.00	6.51	2.70
MDP0000401140	AT1G78080.1	related to AP2.4 (RAP2.4)	162.41	7.34	163.32	7.35	332.19	8.38	188.96	7.56
MDP0000480281	AT4G15800.1	ralf-like 33 (RALFL33)	0.46	-1.12	2.12	1.08	3.78	1.92	3.70	1.89
MDP0000593523	AT4G05390.1	root FNR 1 (RFNR1)	15.60	3.96	14.01	3.81	29.98	4.91	39.19	5.29
MDP0000635733	AT4G33270.1	Transducin family protein / WD-40 repeat	3.31	1.73	6.82	2.77	7.48	2.90	4.77	2.25

		family protein (CDC20.1)								
MDP0000719154	AT1G71040.1	Cupredoxin superfamily protein (LPR2)	3.60	1.85	1.22	0.28	0.53	-0.92	0.59	-0.77
MDP0000934357	AT5G17520.1	root cap 1 (RCP1)	0.81	-0.31	0.49	-1.03	1.18	0.24	1.26	0.33
MDP0000488361	AT5G67400.1	root hair specific 19 (RHS19)	149.41	7.22	223.97	7.81	443.86	8.79	749.03	9.55
MDP0000147890	AT5G66350.1	Lateral root primordium (LRP) protein-related (SHI)	0.20	-2.35	1.81	0.86	4.19	2.07	2.96	1.57
MDP0000132549	AT4G15800.1	ralf-like 33 (RALFL33)	25.18	4.65	45.01	5.49	48.00	5.58	62.23	5.96
MDP0000171430	AT5G12330.4	Lateral root primordium (LRP) protein-related (LRP1)	3.34	1.74	20.55	4.36	30.95	4.95	42.61	5.41
MDP0000147890	AT5G66350.1	Lateral root primordium (LRP) protein-related (SHI)	0.20	-2.35	1.81	0.86	4.19	2.07	2.96	1.57
MDP0000480281	AT4G15800.1	ralf-like 33 (RALFL33)	0.46	-1.12	2.12	1.08	3.78	1.92	3.70	1.89
MDP0000267637	AT4G15800.1	ralf-like 33 (RALFL33)	1.02	0.03	8.47	3.08	7.01	2.81	3.86	1.95

Table S6. Cytokinin-, ethylene-, brassinolide-, jasmonic acid-, abscisic acid-, and wound induction-related genes selected from among the differentially expressed genes in stem cuttings of 'T337' apple rootstocks during the formation of adventitious roots. The colors in every other column indicate different gene expression levels.

Apple genes Identification	Arabidopsis Homolog	Annotation	S1		S2		S3		S4	
			FPKM	Log2 (FPKM)	FPKM	Log2 (FPKM)	FPKM	Log2 (FPKM)	FPKM	Log2 (FPKM)
**CK related**										
MDP0000308379	AT4G27950.1	cytokinin response factor 4 (CRF4)	4.98	2.32	5.52	2.47	10.59	3.40	8.85	3.15
MDP0000238100	AT5G21482.1	cytokinin oxidase 7 (CKX7)	20.53	4.36	9.84	3.30	10.03	3.33	11.70	3.55
MDP0000223673	AT2G41510.1	cytokinin oxidase/dehydrogenase 1 (AT2G41510)(CKX)	1.24	0.31	0.22	-2.21	0.39	-1.37	0.07	-3.82
MDP0000276711	AT2G17820.1	histidine kinase 1 (AHK1)	11.43	3.51	6.81	2.77	2.95	1.56	0.71	-0.49
MDP0000190038	AT4G26150.1	cytokinin-responsive gata factor 1 (GATA22/GATA1)	6.09	2.61	3.05	1.61	1.42	0.50	0.77	-0.38
MDP0000013380	AT3G63110.1	isopentenyltransferase 3 (IPT3)	10.45	3.39	3.85	1.94	1.97	0.98	0.46	-1.11
MDP0000220668	AT5G19040.1	isopentenyltransferase 5 (IPT5)	0.31	-1.68	0.16	-2.61	2.20	1.14	3.18	1.67
MDP0000189484	AT1G68460.1	isopentenyltransferase 1 (IPT1-2)	0.16	-2.61	0.28	-1.81	1.47	0.55	3.33	1.74
MDP0000286203	AT1G68460.1	isopentenyltransferase 1 (IPT1)	0.41	-1.28	0.35	-1.52	2.45	1.29	1.15	0.21
MDP0000172464	AT5G66320.1	GATA transcription factor 5 (GATA5)	13.17	3.72	9.48	3.24	5.97	2.58	3.15	1.66
MDP0000220668	AT5G19040.1	isopentenyltransferase 5 (IPT5)	0.31	-1.68	0.16	-2.61	2.20	1.14	3.18	1.67
**Ethylene related**										
MDP0000787281	AT4G17500.1	ethylene responsive element binding factor 1 (ERF-1)	82.31	6.36	38.32	5.26	41.33	5.37	55.35	5.79
MDP0000517257	AT4G17500.1	ethylene responsive element binding factor 1 (ERF-1)	33.00	5.04	33.30	5.06	41.85	5.39	65.20	6.03
MDP0000226115	AT4G17500.1	ethylene responsive element binding factor 1 (ERF-1)	10.87	3.44	4.55	2.19	1.80	0.85	4.00	2.00
MDP0000791464	AT5G47230.1	ethylene responsive element binding factor 5 (ERF5)	66.60	6.06	67.54	6.08	134.61	7.07	112.16	6.81
MDP0000189033	AT2G44840.1	ethylene-responsive element binding factor 13 (ERF13)	0.01	-6.64	0.06	-3.96	9.41	3.23	5.15	2.37
MDP0000178398	AT2G44840.1	ethylene-responsive element binding factor 13 (ERF13)	0.01	-6.64	0.00		0.13	-2.91	1.99	0.99
MDP0000848905	AT3G16770.1	ethylene-responsive element binding Protein (EBP,ERF72,RAP2.3)	176.30	7.46	137.26	7.10	179.16	7.49	333.83	8.38
MDP0000133485	AT2G44840.1	ethylene-responsive element binding factor 13 (ERF13)	0.15	-2.74	0.08	-3.72	1.42	0.50	1.62	0.70
MDP0000855671	AT3G23240.1	ethylene response factor 1 (ERF1)	1.43	0.52	0.28	-1.82	1.27	0.34	3.77	1.91
MDP0000235313	AT3G23240.1	ethylene response factor 1 (ERF1)	11.11	3.47	2.78	1.48	2.03	1.02	0.87	-0.19
MDP0000127134	AT3G23240.1	ethylene response factor 1 (ERF1)	26.29	4.72	11.79	3.56	7.54	2.91	5.16	2.37
MDP0000195885	AT1G05010.1	ethylene-forming enzyme (EFE)	60.46	5.92	10.67	3.41	11.67	3.54	6.12	2.61
MDP0000200737	AT1G05010.1	ethylene-forming enzyme (EFE2)	28.92	4.85	58.63	5.87	29.95	4.90	26.70	4.74
MDP0000251295	AT1G05010.1	ethylene-forming enzyme (EFE1)	65.13	6.03	25.32	4.66	7.95	2.99	3.95	1.98
MDP0000200896	AT2G19590.1	ACC oxidase 1 (ACO1)	32.61	5.03	92.73	6.54	131.73	7.04	265.95	8.06
**GA related**										
MDP0000161181	AT1G02400.1	gibberellin 2-oxidase 6 (GA2OX4)	1.90	0.92	10.94	3.45	12.91	3.69	20.00	4.32
MDP0000269990	AT4G21200.1	gibberellin 2-oxidase 8 (GA2OX8)	2.72	1.44	0.62	-0.69	0.27	-1.86	0.10	-3.39
MDP0000161181	AT1G02400.1	gibberellin 2-oxidase 6 (GA2OX6)	1.90	0.92	10.94	3.45	12.91	3.69	20.00	4.32
MDP0000145827	AT1G30040.1	gibberellin 2-oxidase (GA2OX2)	16.94	4.08	6.29	2.65	3.75	1.91	0.46	-1.14
MDP0000247490	AT1G30040.1	gibberellin 2-oxidase (GA2OX2-2)	2.36	1.24	1.47	0.56	0.68	-0.56	1.05	0.07
MDP0000381531	AT1G15550.1	gibberellin 3-oxidase 1 (GA3OX1)	1.09	0.12	0.36	-1.49	1.76	0.81	2.26	1.18
MDP0000735747	AT1G02400.1	gibberellin 2-oxidase 6 (GA2OX6)	3.62	1.85	10.70	3.42	5.94	2.57	4.43	2.15
MDP0000185333	AT1G78440.1	Arabidopsis thaliana gibberellin 2-oxidase 1 (GA2OX1-1)	5.20	2.38	0.69	-0.53	4.35	2.12	3.01	1.59
MDP0000137705	AT1G78440.1	Arabidopsis thaliana gibberellin 2-oxidase 1 (GA2OX1)	42.86	5.42	13.58	3.76	24.91	4.64	45.41	5.51
MDP0000212045	AT1G74670.1	Gibberellin-regulated family protein (AT1G74670)	70.82	6.15	341.15	8.41	429.61	8.75	1207.91	10.24
MDP0000126347	AT1G74670.1	Gibberellin-regulated family protein (AT1G74670)	0.01	-6.64	0.16	-2.63	5.31	2.41	3.74	1.90
MDP0000366256	AT5G59845.1	Gibberellin-regulated family protein (AT5G59845)	1.56	0.64	0.76	-0.40	0.74	-0.43	0.12	-3.07

MDP0000150771	AT5G59845.1	Gibberellin-regulated family protein (AT5G59845)	0.32	-1.62	0.01	-6.64	1.24	0.32	0.01	-6.64
MDP0000269551	AT5G14920.1	Gibberellin-regulated family protein (AT5G14920)	57.32	5.84	24.93	4.64	18.73	4.23	8.71	3.12
MDP0000901967	AT5G14920.1	Gibberellin-regulated family protein (AT5G14920)	14.07	3.82	44.03	5.46	333.97	8.38	666.28	9.38
**JA related**										
MDP0000301927	AT5G20900.1	jasmonate-zim-domain protein 12 (JAZ12)	51.81	5.70	20.08	4.33	46.52	5.54	33.55	5.07
MDP0000565690	AT1G72450.1	jasmonate-zim-domain protein 6 (JAZ26)	61.79	5.95	51.17	5.68	81.70	6.35	92.02	6.52
MDP0000901967	AT5G14920.1	jasmonate-zim-domain protein 12 (JAZ12)	14.07	3.82	44.03	5.46	333.97	8.38	666.28	9.38
MDP0000173535	AT2G34600.1	jasmonate-zim-domain protein 7 (JAZ7)	0.01	-6.64	0.01	-6.64	0.01	-6.64	1.97	0.98
**ABA related**										
MDP0000120164	AT5G53160.2	regulatory components of ABA receptor 3 (PYL8,RCAR3)	10.01	3.32	8.87	3.15	4.72	2.24	4.82	2.27
**BR related**										
MDP0000151563	AT4G35230.1	BR-signaling kinase 1 (BSK1)	0.49	-1.04	0.12	-3.04	0.50	-1.00	0.00	-18.47
MDP0000286141	AT3G30180.1	brassinosteroid-6-oxidase 2 (BR6OX2)	0.50	-0.99	1.33	0.42	5.02	2.33	6.46	2.69
**Wd-Id related**										
MDP0000275128	AT4G10270.1	Wound-responsive family protein (Wd-Id-1)	15.93	3.99	7.58	2.92	10.77	3.43	199.14	7.64
MDP0000794258	AT4G10265.1	Wound-responsive family protein (Wd-Id-2)	126.24	6.98	51.78	5.69	59.57	5.90	561.86	9.13
MDP0000732626	AT3G07230.1	wound-responsive protein-related (Wd-Id-3)	32.18	5.01	101.46	6.66	272.62	8.09	195.73	7.61
MDP0000836784	AT4G10265.1	Wound-responsive family protein (Wd-Id-4)	32.79	5.04	20.98	4.39	47.14	5.56	296.89	8.21
MDP0000171638	AT4G10270.1	Wound-responsive family protein (Wd-Id-5)	1.38	0.46	0.62	-0.68	1.44	0.52	77.88	6.28
MDP0000836785	AT4G10265.1	Wound-responsive family protein (Wd-Id-6)	0.77	-0.38	0.96	-0.06	3.23	1.69	17.76	4.15
MDP0000564079	AT4G10265.1	Wound-responsive family protein (Wd-Id-7)	9.83	3.30	5.54	2.47	10.90	3.45	198.30	7.63
MDP0000228919	AT4G28240.1	Wound-responsive family protein (Wd-Id-8)	47.13	5.56	27.49	4.78	16.86	4.08	6.54	2.71
**Pyruvic acid analogues related**										
MDP0000228470	AT2G38310.1	PYR1-like 4 (PYL4)	43.97	5.46	32.00	5.00	52.33	5.71	81.55	6.35
MDP0000270731	AT2G38310.1	PYR1-like 4 (PYL4)	17.06	4.09	13.06	3.71	33.75	5.08	29.83	4.90
MDP0000147358	AT2G26040.1	PYR1-like 2 (PYL2)	0.67	-0.57	0.24	-2.06	5.39	2.43	10.91	3.45
MDP0000437033	AT4G26080.1	Protein phosphatase 2C family protein (ABI1)	23.67	4.56	25.21	4.66	27.98	4.81	11.79	3.56

Table S7. Sugar-related genes selected from among the differentially expressed genes in stem cuttings of 'T337' apple rootstocks during the formation of adventitious roots. The colors in every other column indicate different gene expression levels.

Apple genes Identification	Arabidopsis Homolog	Annotation	S1		S2		S3		S4	
			FPKM	Log2 (FPKM)	FPKM	Log2 (FPKM)	FPKM	Log2 (FPKM)	FPKM	Log2 (FPKM)
**Sucrose/Pectin biosynthesis and metabolism process**										
MDP0000227289	AT4G20050.1	Pectin lyase-like superfamily protein (QRT3)	3.13	1.65	1.13	0.18	1.40	0.49	0.45	-1.17
MDP0000250070	AT3G43190.1	sucrose synthase 4 (SUS4-3)	38.31	5.26	108.78	6.77	65.62	6.04	70.98	6.15
MDP0000872262	AT3G43190.1	sucrose synthase 4 (SUS4)	0.44	-1.18	1.11	0.15	5.53	2.47	6.32	2.66
MDP0000288684	AT4G10120.1	Sucrose-phosphate synthase family protein (ATSPS4F)	8.26	3.05	6.19	2.63	2.38	1.25	1.53	0.62
MDP0000132527	AT3G43190.1	sucrose synthase 4 (SUS4)	1.03	0.05	1.32	0.40	4.22	2.08	4.23	2.08
**Cellulose biosynthesis and metabolism process**										
MDP0000185368	AT2G21770.1	cellulose synthase A9 (CESA9)	9.43	3.24	9.46	3.24	16.59	4.05	27.99	4.81
MDP0000792906	AT2G21770.1	cellulose synthase A9 (CESA9)	4.30	2.10	5.73	2.52	8.48	3.08	9.59	3.26
MDP0000572242	AT4G07960.1	Cellulose-synthase-like C12 (CSLC12)	11.21	3.49	13.15	3.72	21.05	4.40	34.85	5.12
MDP0000553959	AT4G32410.1	cellulose synthase 1 (CESA1,RSW1)	21.41	4.42	24.54	4.62	37.51	5.23	53.74	5.75
MDP0000480237	AT5G64740.1	cellulose synthase 6 (CESA6,E112,IXR2,PRC1)	4.49	2.17	4.53	2.18	6.03	2.59	10.74	3.42
MDP0000128267	AT4G32410.1	cellulose synthase 1 (CESA1,RSW1)	30.88	4.95	33.91	5.08	51.55	5.69	69.63	6.12
MDP0000470441	AT5G17420.1	Cellulose synthase family protein (CESA7,IRX3,MUR10)	0.00	-13.02	0.03	-5.06	0.54	-0.89	0.04	-4.77
MDP0000448752	AT5G05170.1	Cellulose synthase family protein (CESA7,IRX3,MUR10)	45.16	5.50	50.19	5.65	55.36	5.79	79.61	6.31
MDP0000263736	AT5G22740.1	cellulose synthase-like A02 (CSLA02,CSLA2)	16.88	4.08	46.99	5.55	45.69	5.51	78.64	6.30
MDP0000183140	AT3G28180.1	Cellulose-synthase-like C4 (CSLC04,CSLC4)	1.79	0.84	3.61	1.85	6.13	2.62	15.34	3.94
**other**										
MDP0000181206	AT2G19860.1	hexokinase 2 (HXK2)	24.27	4.60	15.69	3.97	24.11	4.59	24.50	4.61
MDP0000940086	AT3G18830.1	polyol/monosaccharide transporter 5 (PMT5)	39.10	5.29	39.42	5.30	22.08	4.46	10.28	3.36
MDP0000755167	AT3G18830.1	polyol/monosaccharide transporter 5 (PMT5)	95.09	6.57	59.49	5.89	48.05	5.59	35.41	5.15
MDP0000175450	AT3G18830.1	polyol/monosaccharide transporter 5 (PMT5)	1.94	0.96	1.08	0.11	6.25	2.64	7.52	2.91
MDP0000688348	AT3G18830.1	polyol/monosaccharide transporter 5(PMT5)	38.73	5.28	19.54	4.29	7.00	2.81	1.79	0.84

Table S8. Enzyme-related genes selected from among the differentially expressed genes in stem cuttings of 'T337' apple rootstock during the formation of adventitious roots. The colors in every other column indicate different gene expression levels.

Apple genes Identification	Arabidopsis Homolog	Annotation	S1		S2		S3		S4	
			FPKM	Log2 (FPKM)	FPKM	Log2 (FPKM)	FPKM	Log2 (FPKM)	FPKM	Log2 (FPKM)
**Biological enzyme related**										
MDP0000602139	AT4G01250.1	WRKY family transcription factor (WRKY)	2.48	1.31	0.49	-1.03	2.26	1.17	2.21	1.14
MDP0000294667	AT5G08640.1	flavonol synthase 1 (FLS1)	3.84	1.94	0.84	-0.25	1.54	0.63	0.00	-21.03
MDP0000302980	AT3G17390.1	S-adenosylmethionine synthetase family protein (SAMS3)	33.20	5.05	36.95	5.21	32.94	5.04	22.01	4.46
MDP0000277049	AT2G22780.1	peroxisomal NAD-malate dehydrogenase 1 (PMDH1)	3.24	1.69	0.56	-0.85	0.96	-0.05	0.23	-2.14
MDP0000602139	AT4G01250.1	WRKY family transcription factor (WRKY22)	2.48	1.31	0.49	-1.03	2.26	1.17	2.21	1.14
